# Cryo-EM snapshots of NMDA receptor activation illuminate sequential rearrangements

**DOI:** 10.1126/sciadv.adx4647

**Published:** 2025-09-24

**Authors:** Jamie A. Abbott, Junhoe Kim, Beiying Liu, Gabriela K. Popescu, Eric Gouaux, Farzad Jalali-Yazdi

**Affiliations:** ^1^ Department of Biochemistry, Jacobs School of Medicine and Biomedical Sciences, University at Buffalo, SUNY, Buffalo, NY 14203, USA.; ^2^ Vollum Institute, Oregon Health and Science University, 3232 SW Research Drive, Portland, OR 97239, USA.; ^3^ Howard Hughes Medical Institute, Oregon Health and Science University, 3232 SW Research Drive, Portland, Oregon 97239, USA.

## Abstract

Canonical *N*-methyl-d-aspartate receptors (NMDARs) are glutamate-gated ion channels with critical roles in the development and function of the nervous system. The excitatory currents they produce reflect stochastic transitions between multiple agonist-bound closed- and open-pore states. We leveraged the intrinsically high open probability (*P*_o_) of NMDARs composed of GluN1 and GluN2A subunits, together with judiciously chosen mutants and ligands, to achieve conditions in which receptors had a *P*_o_ near unity. Using single-particle cryo–electron microscopy (cryo-EM), we captured three activated receptor states, each with distinct conformations of the gate-forming M3 helices. Separately, we carried out single-channel electrophysiology, together with statistical modeling, to relate the cryo-EM structures to the gating reaction. NMDAR channel opening involves bending of the pore-forming M3 helices to produce a transient open-channel conformation, subsequently stabilized by new interactions between the D2-M3 linkers with the pre-M1 helices and the pre-M4 loops, to yield the stable open channel.

## Teaser

Opening of the NMDA receptor involves bending of the helices lining the ion channel gate.

## INTRODUCTION

Canonical *N*-methyl-d-aspartate receptors (NMDARs) are glutamate-gated glycine-dependent ion channels abundant in the central nervous system (*1*, *2*). Their activation results in excitatory currents that underlie synaptic plasticity, cognitive integration, and memory formation (*3*). Mutations in NMDAR-encoding genes result in severe neuropsychiatric disorders and have been implicated in chronic pain syndromes and neurodegenerative conditions (*4*–*6*). To design therapeutic interventions that target the NMDARs, it is necessary to delineate the sequence of conformational changes that constitutes their activation pathway.

NMDARs are large transmembrane proteins that assemble as dimers of heterodimers (*7*–*11*). Each heterodimer consists of an obligatory GluN1 subunit plus a homologous GluN2 or GluN3 subunit, such that functional receptors comprise two GluN1 and two GluN2/3 subunits (*1*). The glycine-binding GluN1 subunit is ubiquitously expressed, whereas the four glutamate-binding GluN2 (A-D) and two glycine-binding GluN3 (A-B) subunits have regulated expression during development and differential distribution in adults (*12*). Upon receptor assembly, homologous domains in each subunit form four distinct layers, known as the amino-terminal (ATD), ligand-binding (LBD), transmembrane (TMD), and carboxy-terminal (CTD) domains. The LBD, which harbors binding sites for the obligatory co-agonists glutamate and glycine, and the TMD, which forms the ion-conducting pore, are required for receptor expression and function. In contrast, the ATD and CTD are involved in assembly, trafficking, localization, and modulation. The TMD consists of three transmembrane helices (M1, M3, and M4) and a short re-entrant loop that includes the M2 helix. The M3 helices line the outer portion of the pore and their intersection, across the conserved SYTANLAAF sequence, forms an agonist-controlled barrier to ion permeation. The pre-M1 helices and pre-M4 linkers form the outer gating ring. The inner portion of the pore is lined by re-entrant M2 helices, whose apex forms the selectivity filter and a binding site for pore blockers such as Mg^2+^, MK-801, and ketamine (*1*, *13*–*15*).

The reaction mechanism of NMDARs is complex and incompletely understood (*16*). Agonists binding to the LBD of resting receptors initiate the activation reaction. Thus primed, receptors can undergo mutually exclusive gating or desensitization reactions. The gating reaction consists of consecutive transitions of primed receptors through quasi-stable closed- and open-pore conformations, whereas the desensitization reactions siphon these receptors away from gating and trap them into long-lived closed-pore conformations (*17*–*19*). Even when fully bound with agonists, most of the NMDAR structures reported to date have closed pores, with straight, closely packed M3 helices (*20*). It is unclear whether these represent primed or desensitized states.

A recently reported agonist-bound GluN1/GluN2B NMDAR structure, in which the two GluN2 M3 helices were bent, resembles an agonist-bound GluA2 AMPA receptor structure with an open pore (*21*, *22*). These studies suggest that breaks in two of the four M3 helices and the resulting movement of the bent M3 helices away from each other are sufficient to open the NMDAR pore. In contrast, open structures of the closely related GluK2 kainate receptors reveal bending of all four M3 helices (*23*). Similarly, a recent single-particle cryo–electron microscopy (cryo-EM) study of GluA2 AMPA receptors heated to physiological temperature immediately prior to flash cooling revealed an open conformation in which all four M3 helices were bent (*24*). Therefore, we suggest that the complete NMDARs gating sequence remains unresolved.

To understand the sequence of events that opens the NMDAR pore, it is necessary to describe the conformations adopted by receptors during gating and the order in which these are occupied. In this study, we report three distinct structures of GluN1/GluN2A NMDAR obtained in conditions where their *P*_o_ is near unity. By comparison with previously determined structures and together with our results from kinetic modeling of fully liganded receptors, we propose that the gating reaction of GluN1/GluN2A NMDARs involves at a minimum these three conformations, referred to here as: primed (closed-pore, all M3 helices straight), engaged (closed-pore, only GluN2 M3 helices bent), and open (open-pore, all four M3 helices bent). Stabilizing the receptor in a stable, highly occupied open state enabled well-defined cryo-EM reconstructions and the accurate positioning of ATD-LBD linkers, LBD-TMD linkers, and TMD, together with bound ligands and lipids.

## RESULTS

### NMDARs can be trapped in multiple activated states

**Fig. 1.**
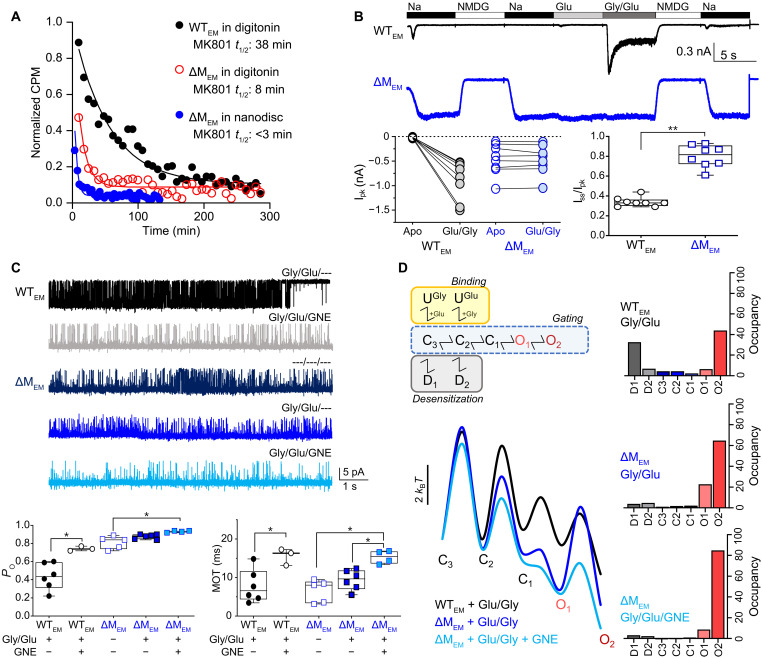
Stabilizing NMDARs in open-pore conformations. (**A**) Time-dependent dissociation of [3H]MK-801 (counts per minute) from WT_EM_ and ΔM_EM_ proteins in the presence of agonists (Gly/Glu), purified in digitonin micelles or reconstituted into lipid-filled nanodiscs (circles), overlayed with single exponential decay functions (lines) fitted to the data. (**B**) Whole-cell currents recorded from HEK293 cells expressing WT_EM_ (black) and ΔM_EM_ (blue) receptors, in the presence of agonists (Gly and Glu) and permeant (Na) or impermeant (NMDG) ions, as indicated. Graphs summarize measured peak current amplitude (*I*_pk_) before and after applying agonists (left), and the extent of current desensitization, expressed as the ratio of the steady-state current relative to the peak current (*I*_ss_/*I*_pk_) (right). Data are from biological replicates. *I*_ss_/*I*_pk_ significance was determined using Student’s unpaired *t* test, ** represent *P* value <0.0001. (**C**) Currents recorded from cell-attached patches containing a single active WT_EM_ (black and gray) or ΔM_EM_ (blue) receptor, with the indicated ligands (Gly, Glu, and GNE-9278) in the recording pipette. Openings are down. Bar graphs (below) summarize *P*_o_ and mean open times (MOTs) calculated for each condition (means ± SD, *n* = 3 to 6). *P*_o_ and MOT significance was determined by one-way ANOVA adjusted with Dunnett’s post hoc test,* represent *P* values <0.01. (**D**) Canonical reaction mechanism (top) illustrates unliganded closed states (U), liganded closed (C) and open (O) gating states, and liganded long-lived closed states (D). Energy landscapes (bottom) for the gating reaction and state occupancies (right) calculated by fitting the canonical model (omitting unliganded states) to pooled data in each condition.

To stabilize open-pore conformations of GluN1/GluN2A receptors, we used a wild-type (WT) construct lacking the unstructured, cytoplasmic CTD (WT_EM_) due to its robust expression in cell culture, ease of biochemical manipulation, and amenity to single-particle cryo-EM analysis (*25*). Nevertheless, when WT_EM_ was reconstituted in digitonin and incubated with agonists, the open channel blocker MK-801 dissociated relatively slowly (*t*_1/2_ ~40 min), suggesting that the receptor has a low *P*_o_ (*26*) (Fig. 1A and table S1). In agreement with this, cryo-EM structural analysis of glycine- and glutamate-bound WT_EM_ at high pH and in the absence of divalent cations, which favor channel opening, revealed a closed-pore conformation [Protein Data Bank (PDB) ID: 6MMP] (*25*).

To identify receptors with higher *P*_o_, we screened combinations of WT_EM_ mutants, ligands, and reconstitution conditions in either digitonin micelles or lipid-filled nanodiscs. A combination of high pH, divalent cation chelation, inclusion of cholesteryl hemisuccinate (CHS), and either the M817V substitution in the GluN2A M4 transmembrane helix (M817V_EM_) (*27*) or deletion of M653 in the GluN2A M3-D2 linker (ΔM_EM_) (*28*) produced receptors with substantially faster MK-801 dissociation rates (Fig. 1A, table S1, fig. S1, A to D). Whole-cell currents recorded from human embryonic kidney (HEK) 293 cells expressing M817V_EM_ or ΔM_EM_ receptors revealed large nondesensitizing currents (*27*) and the ΔM_EM_ receptors were constitutively active (Fig. 1B). To measure unitary amplitudes and *P*_o_ for these receptors, we recorded inward Na^+^ currents from cell-attached patches containing a single active receptor, with or without agonists in the recording pipette (Fig. 1C). Both WT_EM_ and ΔM_EM_ produced currents of similar unitary amplitude to full-length WT GluN1/GluN2A receptors recorded in the same conditions (*29*, *30*) (table S2), indicating that both variants retained an intact permeation pathway. Moreover, ΔM_EM_ receptors were twice as active (*P*_o_ = 0.87 ± 0.03) as WT_EM_ (*P*_o_ = 0.44 ± 0.15), and the positive allosteric modulator (PAM) GNE-9278, but not agonists, further increased their *P*_o_ (0.93 ± 0.01), consistent with previous reports (*31*, *32*). Statistical analyses of open and closed event durations revealed that the higher *P*_o_ of ΔM_EM_ receptors was due to shorter mean closed times (MCTs; 1.4 ± 0.5 ms) than WT_EM_ (9.4 ± 2.4 ms), whereas GNE-9278 increased *P*_o_ by lengthening open times (from 9 ± 3 ms to 15 ± 2 ms) (Fig. 1C and table S2). Further kinetic analyses of these data revealed that, in all recordings, the distributions of closed durations had five components, whereas the distributions of open durations were variable, having between three and four components, as described previously for WT (*16*, *19*) (tables S3 and S4). This analysis indicated that although the conditions we used increased the *P*_o_ to near unity, they did not alter the basic reaction mechanism of these receptors. Next, we used a minimal state model, consisting of five closed and two open states, which has been validated previously for WT receptors (*17*, *33*, *34*), to estimate mean rate constants in the three data sets (fig. S2). The state occupancies and free-energy profiles calculated from these rates illustrate that relative to WT_EM_ receptors, ΔM_EM_ receptors have drastically lower occupancies of desensitized states (Fig. 1D and table S5), consistent with nondesensitizing phenotype observed for these receptors in macroscopic recordings (Fig. 1C). Further, GNE-9278 increased the *P*_o_ of ΔM_EM_, by preferentially increasing the occupancy of the long open-state O_2_ from 0.65 to 0.85 (Fig. 1D and table S5).

**Fig. 2.**
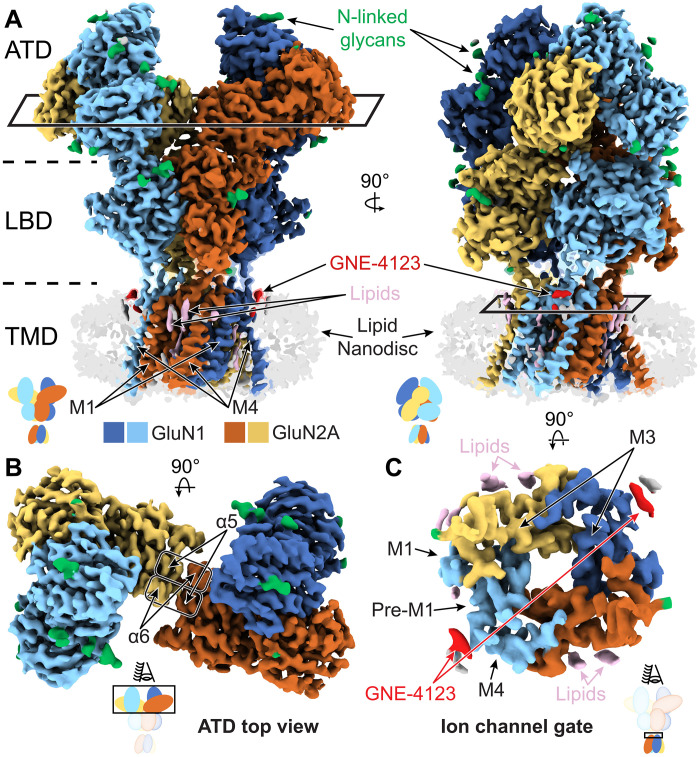
Cryo-EM map of the open state (PDB ID: 9C7C) resolves key functional regions. (**A**) Annotated cryo-EM density of ΔM_EM_ complexed with GNE-4123 obtained in nanodiscs. GluN1 (blue, marine) and GluN2 (brown, yellow). (**B**) Top view of the ATD. (**C**) Top view of the outer gating ring and the M3 helix bundle at the ion channel gate.

Encouraged by these results, we exploited the factors that increased receptor *P*_o_ to elucidate four glycine- and glutamate-bound structures (figs. S3 to S5 and tables S6 and S7). For the cryo-EM experiments, we used GNE-4123, a very close analog of GNE-9278 (fig. S1, C and D) with a higher solubility limit. Both M817V_EM_ with GNE-4123 (PDB ID: 9C7R) and ΔM_EM_ without GNE-4123 (PDB ID: 9C7Q) displayed closed pores. M817V_EM_ with GNE-4123 closely resembled the agonist-bound, two-knuckle symmetric conformation reported for the WT_EM_ receptor (PDB ID: 6MMP) (*25*), both having straight M3 helices. Because M817V_EM_ produced nondesensitizing currents, we hypothesize that the M817V_EM_ with GNE-4123 complex represents one of several “primed” receptor conformations, and when the LBD is fully liganded, the receptor can transition into multiple states, including open conformations (fig. S6). This dataset yielded a higher-resolution map with better resolved transmembrane densities than WT_EM_. Therefore, we used the M817V_EM_ structure as our reference. The structure of the ΔM_EM_ construct in the absence of GNE-4123 revealed a similar ATD conformation and a closed ion channel gate; however, while the GluN1 M3 helices are still straight, the GluN2A M3 helices are bent (figs. S7 to S10). Because ΔM_EM_ produced nondesensitizing currents, with longer mean open times (MOTs), and because its structure revealed bent GluN2A M3 helices and a closed pore, we hypothesize that it represents one of several conformations where the receptors are “engaged” in the gating pathway but not yet open. Last, ΔM_EM_ with GNE-4123, which produced well-resolved complete density maps in both micelles and nanodiscs (PDB IDs: 9C7C and 9C7P) (Fig. 2 and table S6) revealed bent M3 helices for all subunits, with sufficient separation to represent an open pore (Fig. 2C). We therefore designated these two structures as “open” conformations. Both structures are identical at the limit of resolution, with the nanodisc structure having a higher overall resolution. We, therefore, used the nanodisc structure to describe the architecture of the open state.

### ATD-LBD linkers confer subunit-specific modulation

The amino acid sequences of the ATD-LBD linkers vary between NMDAR subunit subtypes and mediate subunit-specific modulation of receptors by their ATDs (*12*, *35*, *36*). The overall arrangement of the ATD layer in all structures reported here is virtually identical to the two-knuckle symmetric conformation of the agonist-bound WT_EM_ receptor (PDB ID: 6MMP) (*25*). This suggests that the ATD adopts an agonist-bound conformation, which can be modified by ligands that bind to the ATD, such as H^+^ and Zn^2+^, but does not change as the receptor transitions toward open-pore conformations (fig. S7, A and B). Consistent with this hypothesis, we found that ΔM_EM_ receptors are resistant to inhibition by Zn^2+^ and H^+^ in whole-cell current recordings (fig. S7, C and D, and table S7).

The open structure of ΔM_EM_ in nanodiscs (3.1 Å) (Fig. 2 and table S6) allowed us to model the backbone and sidechains of the ATD-LBD linkers with confidence and to identify three GluN2A-specific features that confer stability and rigidity on the ATD-LBD interface (fig. S8, A and B). First, a salt bridge between the basic R392 and acidic D402 residues, located near the beginning and end of the linker, creates an 11 residue “stapled” loop. Basic and acidic side chains are conserved at these respective positions in all GluN2 subunits. Second, the loop wraps around Y251 in the ATD, which is conserved in GluN2A and GluN2B subunits. Last, an ATD residue unique to GluN2A, R244, provides further anchoring of the loop via a cation-π interaction with F396 and a salt bridge with D398. This linker-mediated rigidity is absent in GluN2A structures obtained in inhibitory conditions, such as at low pH or in the presence of Zn^2+^ or LBD antagonists (*25*, *37*) and in lower *P*_o_ GluN2B receptors (*16*, *38*). Because the ATD layer acts like a molecular “pincer” on the LBD layer, ensuring that conformational changes within the LBD layer are directed toward the TMD and channel gate, we propose that the well-ordered ATD-LBD linker supports robust coupling between the ATD and LBD of GluN2A subunits. Moreover, the well-defined ATD-LBD linker is likely central to understanding how GluN2A-containing NMDARs have higher *P*_o_ and higher sensitivities to ATD ligands (*12*, *35*, *36*). Under inhibitory conditions, where the GluN2A ATD layer adopts “splayed” conformations, there are fewer contacts between the ATD and LBD layer, thus enabling conformational changes within the LBD layer to result in rearrangements of the LBD that are not transduced to the TMD and, instead, may result in disruption of LBD at the D1-D1 heterodimer interfaces, or at the heterodimer-heterodimer interfaces (*39*).

### In open receptors the LBD layer is closer to the TMD

**Fig. 3.**
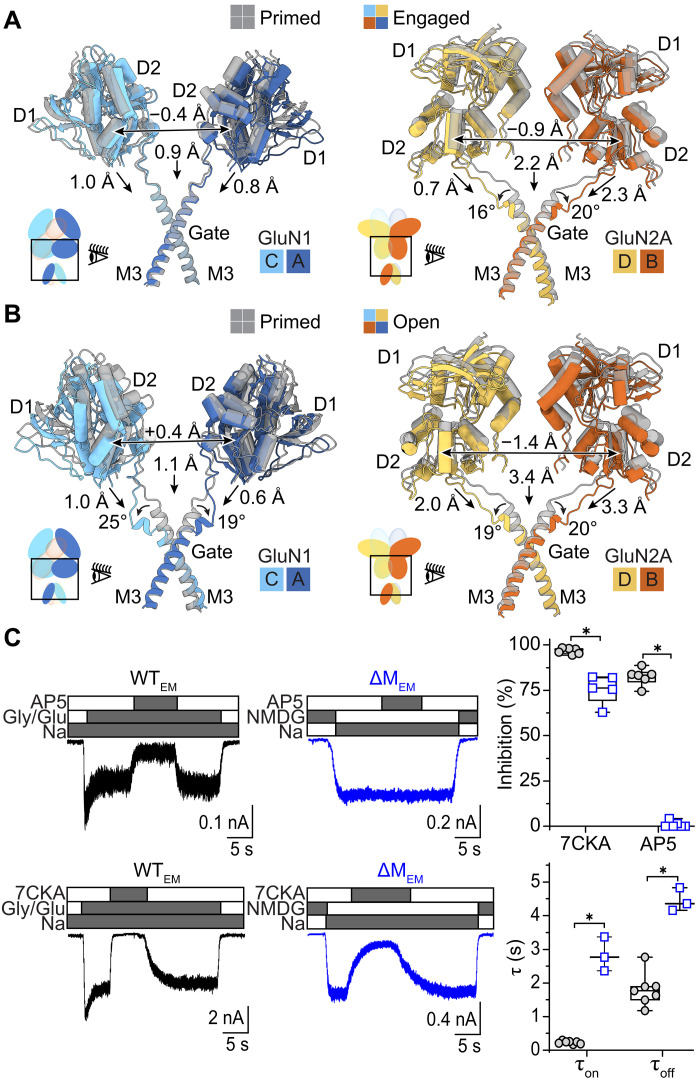
Activation brings the LBD layer closer to the TMD. (**A**) Comparison of the LBD-M3 helix structure of the primed state (gray, PDB ID: 9C7R) with the engaged state (colored, PDB ID: 9C7Q), aligned by the TMD, illustrates movements associated with gate opening. (**B**) Comparison of the LBD-M3 helix structure of the primed state (gray, PDB ID: 9C7R) with the open state in nanodiscs (colored, PDB ID: 9C7C). (**C**) Whole-cell current traces recorded from HEK293 cells expressing WT_EM_ (left) and ΔM_EM_ (middle) constructs in the presence of agonists (Gly and Glu), antagonists specific for GluN1 (7CKA) or GluN2A (AP5), and permeant (Na^+^) or impermeant (NMDG^+^) ions. Bar graphs (right) summarize measured reduction in current (top) and kinetics for ΔM_EM_ relative to WT_EM_ (blue) receptors (biological replicates). *P < 0.0001 (Student’s *t* test).

The LBD layer is by far the most studied portion of the NMDARs (*1*). Consistent with these previous reports (*7*, *25*), we observed closed-clamshell arrangements of the LBDs in all four structures (Fig. 3, A and B). Moreover, the LBD heterodimers are identical to the high-resolution crystal structures of isolated LBDs (*7*) and the distinct interfaces between the LBD heterodimers are the same as those in previous structures of GluN1/GluN2A tetramers (*25*).

Relative to the primed conformation, the D2 lobes of the engaged GluN2A LBD (Fig. 3A) are closer to the TMD by ~2.2 Å and closer to each other by ~0.9 Å. Similar but more subtle changes are apparent in the GluN1 LBDs, resulting in the D2 lobes being closer to the TMD by ~0.9 Å and to each other by 0.4 Å. This observation appears to conflict with the simple model of agonist-dependent gating, where the LBD-TMD linkers pull laterally on the gate to open it. The GluN2A D2 lobes are closer to the TMD by ~3.4 Å and closer to each other by ~1.4 Å in the open conformation compared to the primed conformation (Fig. 3B). Collectively, these conformational changes lead to reductions in the distance between the GluN1 and GluN2A D2 lobes by 1.1 and 1.2 Å in the engaged and open states, both of which lack the M653 residue (fig. S9). These findings are consistent with previously reported decreases in the distance between the GluN1 and GluN2B LBD dimer upon activation (*40*). By contrast, no discernable LBD rotation was observed in this comparison (*41*), likely because all LBDs adopt the same closed-clamshell conformations across the states. Our structures show that gate opening is also a consequence of bending of the M3 helices, which brings the LBD layer closer to the TMD, thus suggesting that the mechanism by which the GluN1 subunits promote ion channel opening—movement toward the TMD—is different from that of GluN2A subunits, which involves tension in the LBD-TMD. Because the GluN2A D2 lobes become progressively closer to each other in more active states of the receptor, our results indicate that shorter distances between opposing GluN2A D2 lobes and closer proximity of D2 lobes to the TMD represent bona fide conformational changes that occur during opening. Moreover, because the GluN2A-specific antagonist, d-2-amino-5-phosphonopentanoate (AP5), has no effect on whole-cell currents carried by the ΔM_EM_ construct, our data support the assumption that this mutation mimics glutamate binding to the GluN2A subunit, effectively locking the GluN2A subunits in an agonist-bound conformation. In contrast, the GluN1-specific antagonist, 7-chlorokynurenic acid (7CKA), remains effective at antagonizing ΔM_EM_ currents (Fig. 3C).

### Subunit-specific rearrangements of M3 open the pore

**Fig. 4.**
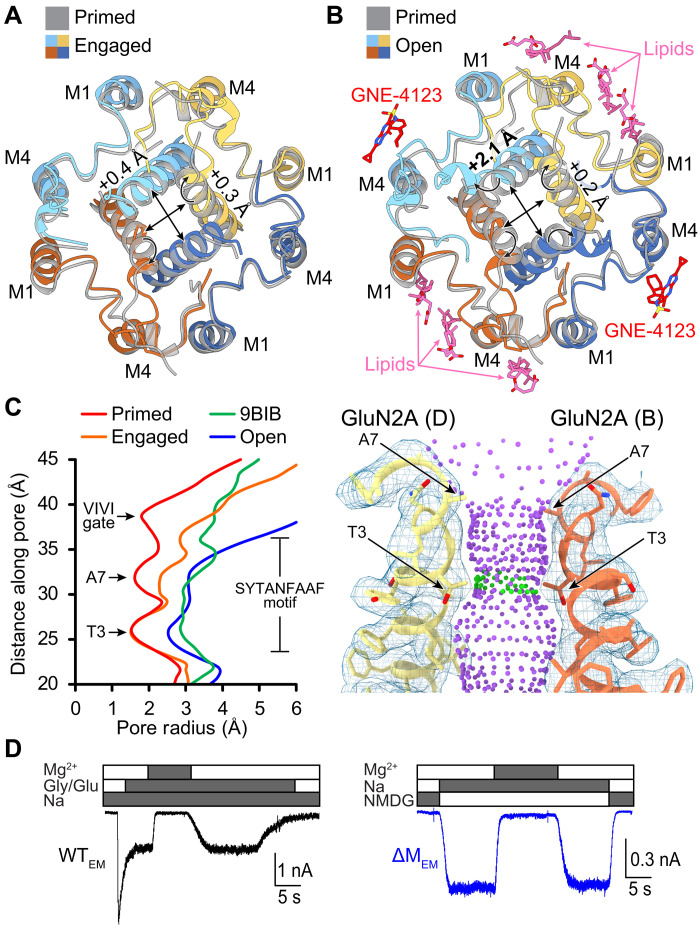
Subunit-specific rearrangements of M3 open the pore. (**A**) Comparison of the gate structures between the engaged state (colored, PDB ID: 9C7Q) and the primed state (gray, PDB ID: 9C7R). (**B**) Comparison of the gate structures between the open state in nanodisc (colored, PDB ID: 9C7C) and the primed state (gray, PDB ID: 9C7R). (**C**) Pore profiles of the open (blue), engaged (orange), and primed (red) states, compared with the open-state GluN1/GluN2B (green, PDB ID: 9BIB). Visualization of ion conduction pathway of the open-state receptor is shown with GluN2A subunits and cryo-EM density (right). Pore radius: red < 1.15 Å < green < 2.3 Å < purple. (**D**) Whole-cell current traces recorded from HEK293 cells expressing WT_EM_ (black, left) or ΔM_EM_ (blue, right) in the presence of agonists (Gly and Glu), open-channel blocker (Mg^2+^), and permeant (Na^+^) or impermeant (NMDG^+^) ions.

The LBD layer communicates with the TMD layer via three short peptide linkers per subunit, which have been resolved in several closed receptor states (*25*, *42*–*44*). However, in conditions that favor more active receptors, both the linker density and the TMD density were poorly resolved (*25*, *43*, *45*). Here, the addition of GNE-4123 allowed us to visualize and model the LBD-TMD linkers and the TMD helices for the engaged and open conformations with confidence (Fig. 4, A and B, and figs. S8C and S10).

In the primed state, all four M3 helices are straight, and the passage of ions is prevented by three constrictions near the extracellular side of the membrane (Fig. 4C) (*28*). We refer to these constrictions as the T3 and A7 gates within the SYTANLAAF motif and the VIVI gate located just above this sequence, at GluN1 V656 and GluN2A I654. In the engaged state, the GluN2A M3 helices are bent, causing the A7 and VIVI gates to become wider by 1.2 and 2.0 Å, respectively. We note that the bending of the GluN2A M3 helices in the engaged state of the ΔM_EM_ structure is similar to the bending of the M3 helices reported in the GluN1/GluN2B open structure (*22*), thus suggesting that it represents a native conformation. However, the GluN1 M3 helices remain straight and the pore diameter at T3 remains sufficiently narrow (3.1 Å) to prevent ion permeation. In contrast, in our open structure, all four M3 helices are bent by 19° to 25°, and the larger pore diameter at T3 (5.1 Å) is consistent with ion permeation. On the basis of these observations, we propose that a stable pore opening of NMDAR requires the bending of all four M3 helices such that the bending of GluN2A M3 opens the VIVI gate and partially opens the A7 gate, whereas bending of GluN1 M3 fully opens the T3 and A7 gates.

In addition to gate widening, M3 bending brings the pre-M1 residues, GluN1 P557 and GluN2A P552, ~1.5 to 3 Å closer to their respective F9 residues in the SYTANLAAF motif, permitting polarized CH-π bonds and van der Waals contacts to form (fig. S11) (*46*, *47*). Another interaction that stabilizes the bent M3 helices is an H-bond between an asparagine on the M4-D2 linker (N812 in GluN1 and N816 in GluN2A) with the backbone carbonyl of the residue after F9 in the SYTANLAAF motif (L655 in GluN1 and M653 in GluN2A), which get closer by ~5.5 Å (fig. S11). These interactions have been proposed to stabilize the open-pore conformation of the receptor, as reported previously (*48*). Despite these robust rearrangements in the “upper” portion of the M3 helices, the “lower” M3 residues that cradle the M2 selectivity filter remain largely unchanged. Consistent with these observations, whole-cell recordings from HEK cells expressing ΔM_EM_ or WT_EM_ reveal similar sensitivity to the pore blocker Mg^2+^ (1 μM). The faster recovery from Mg^2+^ block in ΔM_EM_ receptors is consistent with the higher *P*_o_ of these receptors (Fig. 4D).

### PAMs stabilize the pre-M1 gating ring

**Fig. 5.**
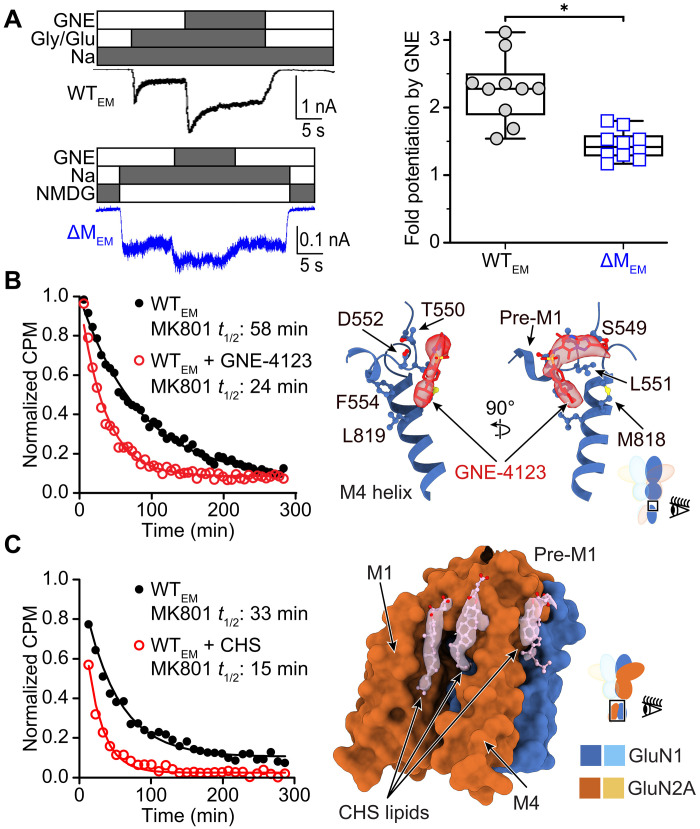
PAMs and bound lipids at the TMD-membrane interface. (**A**) Whole-cell current traces recorded from HEK293 cells expressing WT_EM_ (black, top) and ΔM_EM_ (blue, bottom) constructs in the presence of agonists (Gly and Glu), modulator (GNE-9278), and permeant (Na^+^) or impermeant (NMDG^+^) ions (left), and box plot representing summary of results (right, biological replicates). *P < 0.0001 (Student’s *t* test). (**B**) Time-dependent dissociation of [^3^H]-MK-801 from purified WT_EM_ in the absence (black circles) or presence (red circles) of GNE-4123, overlayed with single exponential decay functions (lines) fitted to the data (left). Position of GNE-4125 and principal contacts with GluN1 pre-M1 TMD helix in the open state in nanodisc (right, PDB ID: 9C7C). (**C**) Time-dependent dissociation of [^3^H]-MK-801 from purified WT_EM_ in the absence (black circles) and presence (red circles) of CHS, overlayed with single exponential decay functions (lines) fitted to the data (left). Position of CHS lipids against M1 and M4 helices of GluN2A, and between M4 helix of GluN2A and M1 helix of GluN1 in the open receptors nanodisc (right).

GNE-9278 is a polycyclic, lipophilic NMDAR PAM that preferentially interacts with glycine- and glutamate-bound receptors to increase current flow through their channels (*32*). Our electrophysiology recordings confirmed that GNE-9278 potentiates whole-cell currents carried by WT_EM_ (Fig. 5A) by increasing the *P*_o_ of unitary openings (Fig. 1C), and that both GNE-9278 and its close variant GNE-4123 increase the MK-801 off-rate in detergent-purified WT_EM_ proteins (Fig. 5B, table S1, and fig. S1, C and D).

In both of our GNE-4123–bound primed and engaged structures, we observed bilateral crescent-shaped densities around the pre-M1 cuff of GluN1 subunits in the vicinity of residues T550, L551, and D552 (Fig. 5B and fig. S12), consistent with prior mutagenesis (*32*). The relatively high resolution of our open structure enabled accurate placement of this GNE-4123 density, showing that it wraps around GluN1 L551, with both ends of the small molecule in close proximity (<4 Å) to the sidechain of L551, positioning the hydrophilic triazolo-pyridone and sulfonamide moieties near the lipid-head groups of the outer leaflet of the membrane (Fig. 5B and fig. S12). GNE-4123 forms two hydrogen bonds with the backbone amide nitrogen atoms of L551 and D552, with distances of 4.0 and 3.8 Å, respectively (fig. S12C). The acidic head group of D552 may also form a weak interaction (distance ~4.4 Å) with the sulfonamide nitrogen of GNE-4123. Consistent with the suggestion that GluN1 L655 is important for the effect of PAMs without being directly involved in their binding (*32*), L655 is only ~3.8 Å away from S553, and this distance increases to 8.6 Å when the receptor gate is closed (fig. S12D).

The conformations of GluN1 in the pre-M1 region are identical in both primed and open structures, consistent with GNE-4123 stabilizing an expanded outer gating ring at the upper leaflet of the membrane and lowering the energy required to break the M3 helix. However, expansion of this outer gating ring is apparently insufficient to fully overcome the barrier to opening the pore, as our GNE-bound M817V_EM_ construct was captured in a primed, closed-pore conformation.

### Lipids surround the transmembrane domain

NMDAR currents are sensitive to some neuroactive steroids and sulfated neurosteroids (*49*), and residues in the TMD have been implicated in these modulatory effects (*50*, *51*). Our cryo-EM sample preparation protocol included CHS, which shares substantial structural similarities with these modulators. When tested in MK-801 dissociation assays, CHS increased the dissociation rate of MK-801 by threefold, consistent with a role as a PAM at the GluN1/GluN2A receptor (table S1 and Fig. 5C). In our open structure in lipid nanodiscs, we resolved several lipid or lipid-like densities near the outer leaflet of the membrane that are consistent with CHS molecules (Fig. 5C). Two of these densities are located between the M1 and M4 helices of each GluN2A subunit. A third density represents a molecule that is packed between each of the GluN1 M1 and GluN2A M4 helices, where it forms extensive interactions with GluN2A Y821.

The location of these CHS molecules is consistent with mutagenesis and MD simulations of GluN2B-containing receptors (*51*), in which the four residues with the most notable effect on neurosteroid modulation were at positions equivalent to those in close proximity to the CHS molecules in our GluN2A-containing structure (W558, D815, Y822, and M823). Together, these observations suggest that CHS molecules may function in concert to stabilize the open conformation of the receptor.

## DISCUSSION

**Fig. 6.**
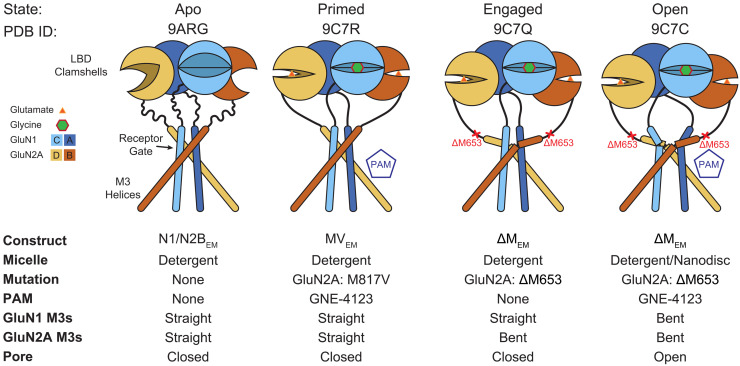
NMDAR gating mechanism. Cartoon of proposed sequence of conformational changes in the LBD clamshells, LBD-TMD linkers, and SYTANLAAF motif, as suggested by functional and structural data.

We have determined four structures of GluN1/GluN2A NMDARs at different points along the activation pathway, revealing how PAMs and lipids or lipid-like molecules stabilize receptor conformations that promote agonist-induced transitions between primed, engaged, and open conformations. Only when all four M3 helices adopt a bent conformation does the receptor transition to the stable open conformation. Our results suggest that agonist-induced gating involves the sequence of conformational changes described in Fig. 6.

Activation of canonical GluN1/GluN2A-D NMDARs not only involves voltage-dependent relief of Mg^2+^ block but also binding of glycine and glutamate to the GluN1 and GluN2A-D subunits, respectively. Nevertheless, even upon reaching this agonist-bound, Mg^2+^-unblocked state, the receptor proceeds through multiple closed-gate conformations. Recently, Chou *et al.* (*22*) reported structures of the GluN1/GluN2B receptor in a variety of apo and ligand-bound states. This study elegantly illustrated the conformational changes that the LBDs undergo upon agonist binding and how a PAM can promote conformations leading to channel opening. However, even in the presence of glycine, glutamate, and a PAM, the most open conformation of the channel gate was associated with only 43% of the total number of particles, the majority occupying a nonactive conformation (*22*). Moreover, the three-dimensional (3D) reconstruction of this conformation yielded density maps to only 3.7 Å resolution, leaving key regions of the structure, including the LBD-TMD linkers, with weak or missing density. Together, this suggests that there is substantial conformational heterogeneity among the particles that is difficult to resolve. The LBDs were well resolved in this GluN1/GluN2B structure and consistent with decades of prior studies showing that agonist binding leads to clamshell closure, but detailed structures of the LBD-TMD linkers were not available. Thus, the question of how conformational changes in the LBDs are propagated through the LBD-TMD linkers to open the channel remained unresolved.

To investigate how clamshell closure gives rise to channel gating, we chose to study the GluN1/GluN2A receptor due to its intrinsically high *P*_o_. We systematically tested experimental conditions, using MK-801 unbinding assays and electrophysiology, to identify factors yielding receptors with the highest *P*_o_. We successfully determined conditions that yielded single-channel recordings with high *P*_o_, including near-unity *P*_o_ for the ΔM mutant combined with a PAM. Cryo-EM reconstructions of receptors in a variety of conditions subsequently enabled us to map the conformational changes from agonist-bound receptors with a closed gate to the fully open state.

Notably, in the open-state conformation, the LBD layer is closer to the TMD and all four M3 helices of the two GluN1 and the two GluN2A subunits bend at the N5 position in the SYTANLAAF motif. This is remarkably similar to a recent open-state structure of the GluA2 AMPA receptor, obtained by heating the sample to physiological temperature immediately before flash cooling, as well as to the open-state conformation of the GluK2 kainate receptor (*23*, *24*). In contrast, only the GluN2B M3 helices are bent in the most open GluN1/GluN2B structure, the GluN1 M3 helices remaining straight (*22*). Because AMPA, kainate, and NMDARs have similar amino acid sequences in the region of the LBD and M3 helices, and because there is evidence for four bent M3 helices associated with open states in all three families, we speculate that the GluN1/GluN2B open state may reflect a less stable open conformation.

Capture of the GluN1/GluN2A receptor in a fully open conformation required addition of a PAM, despite the *P*_o_ of this receptor being near to unity in the absence of PAMs. Presumably, the reduced likelihood of receptors being open in nanodiscs or detergent micelles is a consequence of the absence of a bona fide membrane bilayer. We find that the polycyclic, lipophilic GNE-4123 binds near the likely position of membrane phospholipid head groups around the pre-M1 cuff, perhaps mimicking membrane-receptor interactions. Furthermore, we find strong densities for cholesterol molecules between the M1 and M4 helices, consistent with the membrane and its cholesterol components playing a key role in receptor function.

Our study shows how increasing the tension of the GluN2A M3-D2 linker by deleting M653 (ΔM_EM_ mutant), in combination with addition of lipids and a PAM to better mimic interactions between extracellular regions of the TMD and the lipid bilayer, conspires to fully stabilize glutamate- and glycine-bound GluN1/GluN2A receptors in an open conformation characterized by four bent M3 helices and proximity of the LBD layer to the TMD. These multiple requirements underscore the complexity of NMDAR gating and the extent to which its function can be influenced by agonists, small-molecule modulators, and the lipid bilayer.

## MATERIALS AND METHODS

### Chemicals and reagents

All reagents used for electrophysiology were reagent grade from Sigma-Aldrich, unless otherwise noted. Tissue culture reagents were purchased from Invitrogen (Grand Island, NY).

GNE-9278 was purchased from Sigma-Aldrich, AP5 and 7CKA from Tocris Bioscience, fluorinated octyl maltoside (FOM) and CHS from Anatrace, digitonin from Millipore, brain total lipids from Avanti, desthiobiotin from IBA LifeSciences, MK-801 from Abcam, memantine from Matrix Scientific, and [^3^H]MK-801 from Revvity. GNE-4123 was a gift from Genentech.

The activated and open-pore cryo-EM structures were obtained using GNE-4123, whereas the electrophysiological experiments were performed using GNE-9278. GNE-4123 was a gift from the Genentech Corporation with limited availability. To continue experiments after supplies of GNE-4123 were exhausted, it was necessary to use the closely related and commercially available compound, GNE-9278, instead. The chemical difference between these compounds is minor, with the propyl group in GNE-9278 being changed to a cyclo-propyl group in GNE-4123. We used GNE-4123 in cryo-EM due to slightly improved solubility of the compound compared to GNE-9278 and showed that the compounds have identical effects in increasing the off-rate of MK-801 from purified receptors.

### Experimental model

Sf9 cells [American Type Culture Collection (ATCC) CRL-1711], which originate from female insect tissue, were maintained in Sf-900 III SFM (Thermo Fisher Scientific) at 27°C and used to produce proteins for purification after baculovirus infection.

HEK293 cells (ATCC CRL-1573), were maintained in DMEM supplemented with 10% fetal bovine serum and 1% pen-strep cocktail, at 37°C, in a 5% CO_2_ atmosphere. tsA201 cells (RRID: CVCL-2737), which are derived from HEK293 cells, were cultured in FreeStyle 293 Expression Medium (Sigma-Aldrich) supplemented with 2% fetal bovine serum, at 37°C.

### Construct design

To express WT_EM_ proteins, we used a bicistronic BacMam expression vector described previously (*25*). This vector encodes two CTD-lacking subunits of the *Rattus norvegicus* NMDAR: GluN1-a (Uniprot code: P35439.1), residues 1-847, and GluN2A (Uniprot code: G3V9C5), residues 1-866. Linked to the C terminus, each peptide contains a thrombin recognition site (LVPRGS), a short flexible linker (AAAA), and enhanced green fluorescent protein, followed by either 8xHis-tag on GluN1, or a Strep-tag II on GluN2A.

This bicistronic vector expressing WT_EM_ proteins was further engineered to produce M817V_EM_ proteins by introducing a single residue substitution, GluN2A M817V, or to produce ΔM_EM_ proteins by introducing a single-residue deletion GluN2A ΔM653, using standard site-directed mutagenesis.

### Protein expression for electrophysiology recordings

HEK293 cells (ATCC CRL-1573) were transfected with polyethyleneimine linear, molecular weight 25,000 (Polysciences, Inc.) in a 5:1 ratio (μg PEI:μg DNA). Following transfection, cells were grown 24 to 48 hours in growth medium supplemented with 2 mM Mg^2+^ to prevent NMDAR-mediated cell death.

Before each experiment, cells were washed and covered with phosphate-buffered saline (PBS) and placed on the stage of an inverted microscope. Individual cells were selected visually, based on fluorescence intensity (transfected HEK cells) for patch-clamp recordings.

### Protein expression and purification for structure determination

Proteins were produced and purified as previously reported (*37*, *52*). Virus particles encoding the bicistronic vectors expressing NMDARf subunits were generated and amplified in sf9 cells and were used to infect tsA201 cells. After 10 hours of incubation at 37°C, cultures were treated with 10 mM sodium butyrate and either 0.1 mM memantine or 10 μM MK-801, before shifting them to 30°C. At 36 to 48 hours postinfection, cells were harvested, washed, resuspended in lysis buffer [tris-buffered saline (TBS) supplemented with 2% digitonin, 1 mM CHS, and protease inhibitors], and incubated 90 min at 4°C. After centrifugation (185K RCF) and filtration (0.45 μm), the supernatant was passed over Strep-Tactin Sepharose resin (IBA LifeSciences). The protein fraction was eluted per manufacturer’s instructions, concentrated and further purified using Superose 6 Increase 10/300 GL size exclusion chromatography (SEC) column (Cytiva). Peak fractions were combined, concentrated, and used for further experiments.

### Nanodisc reconstitution

To create a solution of brain total lipids solubilized in detergent, equal volumes of a solution of 2% digitonin and 0.2% CHS in 1× TBS were combined with a homogenized solution (10 mg/ml) of brain total lipids and incubated for 1 hour at 4°C. For reconstitution in nanodiscs, 2 mg of affinity-purified NMDAR (pre-SEC step) at 2 mg/ml was combined with sixfold molar excess of MSP1E3D1 membrane scaffold protein (*53*) and 1 mg of digitonin-solubilized brain total lipids (~400× molar excess, also containing 0.2 mg CHS) and incubated by end-over-end rotation at 4°C for 1 hour. To remove the detergent, 300-mg Bio-Beads SM-2 resin (Bio-Rad), washed with methanol and deionized water per manufacturer’s instructions, was added per 500 μl of solution, and incubated end-over-end at 4°C for 2 hours. The Bio-Beads in the reconstitution solution were exchanged with fresh beads after 2 hours and the process was repeated twice. The solution was then incubated overnight with fresh Bio-Beads. The sample was spun at 10,000 rpm at 4°C in a bench-top centrifuge for 10 min, pelleting nonincorporated lipids. The supernatant was further clarified by filtration, concentrated, and run on a SEC column in 1× TBS supplemented with 2 mM EDTA. The peak fraction was collected and analyzed via SDS–polyacrylamide gel electrophoresis and fluorescent size exclusion chromatography (FSEC), in a detergent-free buffer, to validate reconstitution into nanodiscs.

### Scintillation proximity assay

Scintillation proximity assays (SPAs) were set up using 40 nM purified receptor in 1× TBS (pH 8.0) supplemented with 0.1% digitonin, 2 mM EDTA, YSI-WGA beads (0.5 mg/ml; Revvity), and 100 nM [^3^H]MK-801 per well. If CHS was used as an additive, the protein was in a buffer containing 200 μM CHS and the assay buffer also contained the same concentration of CHS. The final volumes were 100 μl per well. For the nanodisc samples, no detergent was used in the assay solutions. Because of the high levels of background detected in the absence of detergent in the nanodisc samples, the beads were switched to copper HIS-tag YSI beads (Revvity), EDTA was omitted, and bovine serum albumin (10 mg/ml) was added to reduce nonspecific binding. The plate was placed inside a Microbeta TriLux 1450 LSC plate reader (PerkinElmer) and read at the rate of 20 s per well. After allowing for [^3^H]MK-801 binding to reach saturation, memantine to a final concentration of 1 mM was added to each sample to prevent the re-binding of radiolabeled ligand, and the plate was placed back in the plate reader to measure the off-rate of MK-801. Data were used to fit a single-site binding model.

### Cryo-EM sample, grid preparation and data collection

Droplets of protein solution (~4 μl at 3 to 4 mg/ml), supplemented with agonists and modulators as specified, were placed on mesh grids (Quantifoil 2/1 Au 300) and were glow-discharged for 1 min at 15 mA with the carbon side facing up. The grid was then blotted for 3 s at 18°C (Vitrobot, 100% humidity), and flash-frozen in liquid ethane. For the nanodisc experiments, in addition to agonists (1 mM Gly and 1 mM Glu) and GNE-4123 (0.1 mM), samples also contained FOM (0.1 mM).

Data collections were carried out at either the Pacific Northwest Center for CryoEM (PNCC) or the National Center for CryoEM Access and Training (NCCAT) at New York Structural Biology Center (NYSBC) using a 300-kV FEI Titan Krios equipped with an energy filter. At NYSBC, images were collected using a Gatan K2 direct electron detector in super-resolution mode at unbinned pixel size of 0.523 Å/pixel using Leginon. At PNCC, images were collected using a Gatan K3 sensor at unbinned pixel size of 0.413 or 0.4115 Å per pixel using serialEM^8^ with a typical defocus range of −0.8 to −2.0 μm.

### Cryo-EM data and image processing

Image stacks were aligned to correct for beam-induced motion during recording and were dose-weighted to compensate for radiation damage using MotionCor2 (*54*) or CryoSPARC (version 3) (*55*). The movies were Fourier-space binned 2 × 2 during motion correction to obtain images at physical pixel values, and the contrast transfer function was estimated using Gctf 11 or CryoSPARC CTF estimator (*55*). Template-free automatic particle selection was done using DoG-Picker (*56*) (https://sbgrid.org/software/titles/gautomatch), or CryoSPARC’s template free 2D particle picker (*55*). To generate the initial 3D model, reference-free 2D classification of particles was carried out using CryoSPARC followed by manual inspection, selection of the classes representing NMDARs, and further classification. In general, two rounds of 2D classification were performed. After 2D cleanup of reference-free picked particles, a conservatively selected few classes were used for ab initio generation of the initial 3D model. The generated model was then used for 3D classification of the initial set of picked particles before 2D cleanup. Typically, eight classes were used for this initial cleanup, setting the box size so that the ostensible resolution limit imposed by Nyquist frequency would be 4 to 6 Å. After 3D classification, generally only a single class showed high-resolution features, and this class was then further processed by 3D classification, either with four to eight copies of the same initial model or by generating four to eight models via ab initio generation and using them as initial models for 3D classification in CryoSPARC (*55*). After additional rounds of refinement and further classification, the final particle set for each class was exported to RELION (3.0 beta) (*57*) and further 3D classification and refinement were performed, although this manipulation did not improve resolution or map features.

### Model building and refinement

We used as initial models those we developed previously for WT_EM_ (*25*). Each subunit was split into five parts (R1, R2, D1, D2, and TMD) and the resulting 20 regions were fitted independently as rigid bodies into the respective cryo-EM density maps using UCSF Chimera (*58*). Each model was then manually inspected and corrected in COOT (*59*) and followed by real-space refinement in Phenix (*60*), Fourier shell correlation curves correlating the final models to the cryo-EM maps were generated in Phenix.

### Patch-clamp electrophysiology

Macroscopic currents were recorded with the whole-cell patch-clamp technique as described previously (*61*). In brief, glass electrodes were filled with (intracellular) solutions containing the following: 135 mM CsF, 33 mM CsOH, 2 mM MgCl_2_, 1 mM CaCl_2_, 10 mM HEPES, and 11 mM EGTA, adjusted to pH 7.4 (CsOH). After reaching the whole-cell configuration, the voltage was clamped at −70 mV and cells were superfused with (extracellular) solutions using a lightly pressurized perfusion system that could exchange solutions within 0.3 to 0.5 s (BPS-8, ALA Scientific Instruments, Westbury, NY). Extracellular solutions contained the following: 150 mM NaCl, 2.5 mM KCl, 0.5 mM CaCl_2_, 0.01 mM mM EDTA, and 10 mM HEPES buffer, adjusted to pH 7.2 (NaOH). Glutamate (1 mM), glycine (0.1 mM), GNE-9278, AP5, or 7CKA were added to the extracellular solution, as indicated. In experiments testing the effect of Zn^2+^, the extracellular solution contained the following: 150 mM NaCl, 2.5 mM KCl, 0.25 mM CaCl_2_, 10 mM tricine, 10 nM ZnCl_2,_ and NaOH to pH 7.2. All currents were amplified and low-pass filtered at 2 kHz (Axopatch200B; 4-pole Bessel), sampled at 5 kHz (DigiData 1322A), and written into digital files with pClamp 8 acquisition software (Molecular Devices, Sunnyvale, CA). Current amplitudes for peak (*I*_pk_) and steady-state (*I*_ss_) levels were measured in pClamp. Inhibition kinetics (onset and offset) were measured by fitting exponential functions to the decline (τ_on_) and recovery (τ_off_) of the current, respectively.

Unitary currents were recorded with the cell-attached patch-clamp technique as described previously (*62*). During recordings, cells were bathed in phosphate-buffered saline. Glass electrodes (outer diameter, 1.5 mm; inner diameter 0.86 mm; Sutter Instruments) were fire polished to a final resistance of 12 to 24 megohms. In brief, the electrodes were filled with (extracellular) solutions containing the following: 150 mM NaCl, 2.5 mM KCl, 1 mM EDTA, 10 mM HEPBS [*N*-(2-hydroxyethyl) piperazine-*N*′(4-butanesulfonic acid)], 0.1 mM glycine, 1 mM glutamate, and, where indicated, GNE-9278, adjusted to pH 8 (NaOH). Inward sodium currents were recorded after applying +100 mV through the recording pipette (estimated final membrane-patch potential ~−120 mV). Currents were amplified and low pass filtered at 10 kHz (Axopatch200B; 4-pole Bessel), sampled at 40 kHz (PCI-6229, M Series card, National Instruments, Austin, TX), and written onto computer hard drives with QUB acquisition software (available upon request).

### Data analyses

#### Macroscopic recordings

Macroscopic whole-cell recordings were analyzed using Clampfit (Molecular Devices, Union City, CA) and Origin (Microal Software, Northhampton, MA). Percent inhibition by NMDA modulators was determined by measuring steady-state current amplitudes with (*I*_drug_) and without drug (*I*_ctr_), where each value represents the average current observed

$$\%\;\text{current inhibited}=\left(1+I_{\text{drug}}/I_{\text{ctr}}\right)\times 100$$

$$\%\;\text{current potentiation}=\left(I_{\text{drug}}/I_{\text{ctr}}-1\right)\times 100$$

With Clampfit onset and offset times (τ) were estimated by fitting exponential function to the onset and offset phases of current inhibition and recovery, respectively. To compare onset and offset times, we used one-way analysis of variance (ANOVA) with Tukey’s multiple comparisons test.

### Microscopic recordings

Single channel analyses were done in QUB. In brief, idealization was done with the segmental-*k*-means (SKM) algorithm applied to digitally filtered data (12 kHz) with no imposed dead time. After idealization of each trace, we calculated single-channel current amplitudes and kinetic parameters for each recording: equilibrium *P*_o_, MOT, and MCT.

### Kinetic modeling and free energy calculations

Kinetic modeling was done for data obtained from individual receptors using the maximum-interval likelihood method in QUB software, by fitting user-defined state models to idealized data after imposing a 0.075 ms deadtime. Models of increasing complexity were fitted individually to each data file and the best fitting model was selected on the basis of optimal visual agreement between the predicted probability density function with the data and the maximum of the log-likelihood (LL) function. The optimal number of states was determined according to Akaike criterion requiring the LL to increase by at least 20 units for a state to be considered necessary in the model. The rate constants resulting from the gating models were used to calculate free-energy landscapes according to the relationship Δ*G*0 = −*R*·*T*·ln(Keq), where *R* is the molar gas constant, *T* is the absolute temperature, and Keq is the equilibrium constant for the transition considered. Barrier heights were estimated as *E*† = Δ*G*0 + [10 − ln(kf)]. Energy differences were calculated relative to state C3.

For figures we used GraphPad Prism 10.2.3 software to generate box and whisker plots and compare electrophysiology data. For % inhibition box and whiskers plots displayed in figures, values for individual cells are shown. The box represents the 25th to 75th percentiles, the middle of the box is the median, while whiskers represent the minimum and the maximum values. To evaluate differences in single dose inhibition or % potentiation between WT_EM_ and ∆M_EM_, we used unpaired Student’s *t* tests. To compare single-channel parameters *P*_o_, MCT, and MOT in presence and absence of agonist and/or GNE-9278 we used one-way ANOVA with Tukey’s multiple comparisons test. This work was an exploratory study and the number of cells collected for each experiment was not predetermined before the work was done and as such *P* values reported are to be interpreted as descriptive only.

## References

[1] K. B. Hansen, F. Yi, R. E. Perszyk, H. Furukawa, L. P. Wollmuth, A. J. Gibb, S. F. Traynelis, Structure, function, and allosteric modulation of NMDA receptors. J. Gen. Physiol. 150, 1081–1105 (2018). 30037851 10.1085/jgp.201812032 PMC6080888

[2] K. B. Hansen, L. P. Wollmuth, D. Bowie, H. Furukawa, F. S. Menniti, A. I. Sobolevsky, G. T. Swanson, S. A. Swanger, I. H. Greger, T. Nakagawa, C. J. McBain, V. Jayaraman, C. M. Low, M. L. Dell’Acqua, J. S. Diamond, C. R. Camp, R. E. Perszyk, H. Yuan, S. F. Traynelis, Structure, function, and pharmacology of glutamate receptor ion channels. Pharmacol. Rev. 73, 298–487 (2021). 34753794 10.1124/pharmrev.120.000131 PMC8626789

[3] D. L. Hunt, P. E. Castillo, Synaptic plasticity of NMDA receptors: Mechanisms and functional implications. Curr. Opin. Neurobiol. 22, 496–508 (2012). 22325859 10.1016/j.conb.2012.01.007 PMC3482462

[4] S. F. Traynelis, L. P. Wollmuth, C. J. McBain, F. S. Menniti, K. M. Vance, K. K. Ogden, K. B. Hansen, H. Yuan, S. J. Myers, R. Dingledine, Glutamate receptor ion channels: Structure, regulation, and function. Pharmacol. Rev. 62, 405–496 (2010). 20716669 10.1124/pr.109.002451 PMC2964903

[5] C. Hu, W. Chen, S. J. Meyers, H. Yuan, S. F. Traynelis, Human GRIN2B variants in neurodevelopmental disorders. J. Pharmacol. Sci. 132, 115–121 (2016). 27818011 10.1016/j.jphs.2016.10.002 PMC5125235

[6] J. P. Dupuis, O. Nicole, L. Groc, NMDA receptor functions in health and disease: Old actor, new dimensions. Neuron 111, 2312–2328 (2023). 37236178 10.1016/j.neuron.2023.05.002

[7] H. Furukawa, S. Singh, R. Mancusso, E. Gouaux, Subunit arrangement and function in NMDA receptors. Nature 438, 185–192 (2005). 16281028 10.1038/nature04089

[8] E. Karakas, N. Simorowski, H. Furukawa, Subunit arrangement and phenylethanolamine binding in GluN1/GluN2 NMDA receptors. Nature 475, 249–253 (2011). 21677647 10.1038/nature10180 PMC3171209

[9] C. H. Lee, E. Gouaux, Amino terminal domains of the NMDA receptor are organized as local heterodimers. PLOS ONE 6, e19180 (2011). 21544205 10.1371/journal.pone.0019180 PMC3081335

[10] E. Karakas, H. Furukawa, Crystal structure of a heteromeric NMDA receptor ion channel. Science 344, 992–997 (2014). 24876489 10.1126/science.1251915 PMC4113085

[11] C. H. Lee, W. Lu, J. Carlisle Michel, A. Goehring, J. Du, X. Song, E. Gouaux, NMDA receptor structures reveal subunit arrangement and pore architecture. Nature 511, 191–197 (2014). 25008524 10.1038/nature13548 PMC4263351

[12] P. Paoletti, C. Bellone, Q. Zhou, NMDA receptor subunit diversity: Impact on receptor properties, synaptic plasticity and disease. Nat. Rev. Neurosci. 14, 383–400 (2013). 23686171 10.1038/nrn3504

[13] X. Song, M. Ø. Jensen, V. Joginin, R. A. Stein, C. H. Lee, H. S. Mchaourab, D. E. Shaw, E. Gouaux, Mechanism of NMDA receptor channel block by MK-801 and memantine. Nature 556, 515–519 (2018). 29670280 10.1038/s41586-018-0039-9 PMC5962351

[14] Y. Zhang, F. Ye, T. Zhang, S. Lv, L. Zhou, D. Du, H. Lin, F. Guo, C. Luo, S. Zhu, Structural basis of ketamine action on human NMDA receptors. Nature 596, 301–305 (2021). 34321660 10.1038/s41586-021-03769-9

[15] J. B. Amin, M. He, R. Prasad, X. Leng, H. X. Zhou, L. P. Wollmuth, Two gates mediate NMDA receptor activity and are under subunit-specific regulation. Nat. Commun. 14, 1623 (2023). 36959168 10.1038/s41467-023-37260-y PMC10036335

[16] G. J. Iacobucci, G. K. Popescu, Kinetic models for activation and modulation of NMDA receptor subtypes. Curr. Opin. Physio. 2, 114–122 (2018). 10.1016/j.cophys.2018.02.002 PMC6029435 29978141

[17] G. Popescu, A. Auerbach, Modal gating of NMDA receptors and the shape of their synaptic response. Nat. Neurosci. 6, 476–483 (2003). 12679783 10.1038/nn1044

[18] T. G. Banke, S. F. Traynelis, Activation of NR1/NR2B NMDA receptors. Nat. Neurosci. 6, 144–152 (2003). 12524545 10.1038/nn1000

[19] C. L. Kussius, G. K. Popescu, Kinetic basis of partial agonism at NMDA receptors. Nat. Neurosci. 12, 1114–1120 (2009). 19648915 10.1038/nn.2361 PMC2739723

[20] C. Zhou, N. Tajima, Structural insights into NMDA receptor pharmacology. Biochem. Soc. Trans. 51, 1713–1731 (2023). 37431773 10.1042/BST20230122 PMC10586783

[21] E. C. Twomey, M. V. Yelshanskaya, R. A. Grassucci, J. Frank, A. I. Sobolevsky, Channel opening and gating mechanism in AMPA-subtype glutamate receptors. Nature 549, 60–65 (2017). 28737760 10.1038/nature23479 PMC5743206

[22] T. H. Chou, M. Epstein, R. G. Fritzemeier, N. S. Akins, S. Paladugu, E. Z. Ullman, D. C. Liotta, S. F. Traynelis, H. Furukawa, Molecular mechanism of ligand gating and opening of NMDA receptor. Nature 632, 209–217 (2024). 39085540 10.1038/s41586-024-07742-0 PMC11376105

[23] S. P. Gangwar, M. V. Yelshanskaya, K. D. Nadezhdin, L. Y. Yen, T. P. Newton, M. Aktolun, M. G. Kurnikova, A. I. Sobolevsky, Kainate receptor channel opening and gating mechanism. Nature 630, 762–768 (2024). 38778115 10.1038/s41586-024-07475-0 PMC11186766

[24] A. K. Mondal, E. Carrillo, V. Jayaraman, E. C. Twomey, temperature sensitive glutamate gating of AMPA-subtype iGluRs. *bioRxiv*, 2024.09.05.611422 (2024); 10.1101/2024.09.05.611422.

[25] F. Jalali-Yazdi, S. Chowdhury, C. Yoshioka, E. Gouaux, Mechanisms for zinc and proton inhibition of the GluN1/GluN2A NMDA receptor. Cell 175, 1520–1532.e15 (2018). 30500536 10.1016/j.cell.2018.10.043 PMC6333211

[26] J. E. Huettner, B. P. Bean, Block of N-methyl-D-aspartate-activated current by the anticonvulsant MK-801: Selective binding to open channels. Proc. Natl. Acad. Sci. U.S.A. 85, 1307–1311 (1988). 2448800 10.1073/pnas.85.4.1307 PMC279756

[27] W. Chen, A. Tankovic, P. B. Burger, H. Kusumoto, S. F. Traynelis, H. Yuan, Functional evaluation of a de novo GRIN2A mutation identified in a patient with profound global developmental delay and refractory epilepsy. Mol. Pharmacol. 91, 317–330 (2017). 28126851 10.1124/mol.116.106781 PMC5363715

[28] M. Ladislav, J. Cerny, J. Krusek, M. Horak, A. Balik, L. Vyklicky, The LiLi motif of M3-S2 linkers is a component of the NMDA receptor channel gate. Front. Mol. Neurosci. 11, 113 (2018). 29681798 10.3389/fnmol.2018.00113 PMC5897735

[29] G. J. Iacobucci, G. K. Popescu, Ca^2+^-dependent inactivation of GluN2A and GluN2B NMDA receptors occurs by a common kinetic mechanism. Biophys. J. 118, 798–812 (2020). 31629478 10.1016/j.bpj.2019.07.057 PMC7036730

[30] D. A. Chopra, K. Sapkota, M. W. Irvine, G. Fang, D. E. Jane, D. T. Monaghan, S. M. Dravid, A single-channel mechanism for pharmacological potentiation of GluN1/GluN2A NMDA receptors. Sci. Rep. 7, 6933 (2017). 28761055 10.1038/s41598-017-07292-8 PMC5537304

[31] D. H. Hackos, J. E. Hanson, Diverse modes of NMDA receptor positive allosteric modulation: Mechanisms and consequences. Neuropharmacology 112, 34–45 (2017). 27484578 10.1016/j.neuropharm.2016.07.037

[32] T. M. Wang, B. M. Brown, L. Deng, B. D. Sellers, P. J. Lupardus, H. J. A. Wallweber, A. Gustafson, E. Wong, M. Volgraf, J. B. Schwarz, D. H. Hackos, J. E. Hanson, A novel NMDA receptor positive allosteric modulator that acts via the transmembrane domain. Neuropharmacology 121, 204–218 (2017). 28457974 10.1016/j.neuropharm.2017.04.041

[33] G. Popescu, A. Robert, J. R. Howe, A. Auerbach, Reaction mechanism determines NMDA receptor response to repetitive stimulation. Nature 430, 790–793 (2004). 15306812 10.1038/nature02775

[34] G. J. Iacobucci, G. K. Popescu, NMDA receptors: Linking physiological output to biophysical operation. Nat. Rev. Neurosci. 18, 236–249 (2017). 28303017 10.1038/nrn.2017.24 PMC5640446

[35] M. Gielen, B. Siegler Retchless, L. Mony, J. W. Johnson, P. Paoletti, Mechanism of differential control of NMDA receptor activity by NR2 subunits. Nature 459, 703–707 (2009). 19404260 10.1038/nature07993 PMC2711440

[36] H. Yuan, K. B. Hansen, K. M. Vance, K. K. Ogden, S. F. Traynelis, Control of NMDA receptor function by the NR2 subunit amino-terminal domain. J. Neurosci. 29, 12045–12058 (2009). 19793963 10.1523/JNEUROSCI.1365-09.2009 PMC2776059

[37] S. Zhu, R. A. Stein, C. Yoshioka, C. H. Lee, A. Goehring, H. S. Mchaourab, E. Gouaux, Mechanism of NMDA receptor inhibition and activation. Cell 165, 704–714 (2016). 27062927 10.1016/j.cell.2016.03.028 PMC4914038

[38] S. A. Amico-Ruvio, G. K. Popescu, Stationary gating of GluN1/GluN2B receptors in intact membrane patches. Biophys. J. 98, 1160–1169 (2010). 20371315 10.1016/j.bpj.2009.12.4276 PMC2849062

[39] M. Tian, D. Stroebel, L. Piot, M. David, S. Ye, P. Paoletti, GluN2A and GluN2B NMDA receptors use distinct allosteric routes. Nat. Commun. 12, 4709 (2021). 34354080 10.1038/s41467-021-25058-9 PMC8342458

[40] N. Tajima, E. Karakas, T. Grant, N. Simorowski, R. Diaz-Avalos, N. Grigorieff, H. Furukawa, Activation of NMDA receptors and the mechanism of inhibition by ifenprodil. Nature 534, 63–68 (2016). 27135925 10.1038/nature17679 PMC5136294

[41] J. B. Esmenjaud, D. Stroebel, K. Chan, T. Grand, M. David, L. P. Wollmuth, A. Taly, P. Paoletti, An inter-dimer allosteric switch controls NMDA receptor activity. EMBO J. 38, e99894 (2019). 30396997 10.15252/embj.201899894 PMC6331725

[42] T. H. Chou, N. Tajima, A. Romero-Hernandez, H. Furukawa, Structural basis of functional transitions in mammalian NMDA receptors. Cell 182, 357–371.e13 (2020). 32610085 10.1016/j.cell.2020.05.052 PMC8278726

[43] H. Wang, S. Lv, D. Stroebel, J. Zhang, Y. Pan, X. Huang, X. Zhang, P. Paoletti, S. Zhu, Gating mechanism and a modulatory niche of human GluN1-GluN2A NMDA receptors. Neuron 109, 2443–2456.e5 (2021). 34186027 10.1016/j.neuron.2021.05.031

[44] N. Tajima, N. Simorowski, R. A. Yovanno, M. C. Regan, K. Michalski, R. Gomez, A. Y. Lau, H. Furukawa, Development and characterization of functional antibodies targeting NMDA receptors. Nat. Commun. 13, 923 (2022). 35177668 10.1038/s41467-022-28559-3 PMC8854693

[45] T. H. Chou, H. Kang, N. Simorowski, S. F. Traynelis, H. Furukawa, Structural insights into assembly and function of GluN1-2C, GluN1-2A-2C, and GluN1-2D NMDARs. Mol. Cell 82, 4548–4563.e4 (2022). 36309015 10.1016/j.molcel.2022.10.008 PMC9722627

[46] B. Leitgeb, G. Toth, Aromatic-aromatic and proline-aromatic interactions in endomorphin-1 and endomorphin-2. Eur. J. Med. Chem. 40, 674–686 (2005). 15935902 10.1016/j.ejmech.2004.10.015

[47] N. J. Zondlo, Aromatic-proline interactions: Electronically tunable CH/pi interactions. Acc. Chem. Res. 46, 1039–1049 (2013). 23148796 10.1021/ar300087y PMC3780429

[48] G. J. Iacobucci, B. Liu, H. Wen, B. Sincox, W. Zheng, G. K. Popescu, Complex functional phenotypes of NMDA receptor disease variants. Mol. Psychiatry 27, 5113–5123 (2022). 36117210 10.1038/s41380-022-01774-6 PMC11963734

[49] M. Park-Chung, F. S. Wu, R. H. Purdy, A. A. Malayev, T. T. Gibbs, D. H. Farb, Distinct sites for inverse modulation of N-methyl-D-aspartate receptors by sulfated steroids. Mol. Pharmacol. 52, 1113–1123 (1997). 9396781 10.1124/mol.52.6.1113

[50] M. K. Jang, D. F. Mierke, S. J. Russek, D. H. Farb, A steroid modulatory domain on NR2B controls N-methyl-D-aspartate receptor proton sensitivity. Proc. Natl. Acad. Sci. U.S.A. 101, 8198–8203 (2004). 15150412 10.1073/pnas.0401838101 PMC419580

[51] B. Hrcka Krausova, B. Kysilov, J. Cerny, V. Vyklicky, T. Smejkalova, M. Ladislav, A. Balik, M. Korinek, H. Chodounska, E. Kudova, L. Vyklicky, Site of action of brain neurosteroid pregnenolone sulfate at the N-methyl-D-aspartate receptor. J. Neurosci. 40, 5922–5936 (2020). 32611707 10.1523/JNEUROSCI.3010-19.2020 PMC7392504

[52] Y. Zhao, S. Chen, C. Yoshioka, I. Baconguis, E. Gouaux, Architecture of fully occupied GluA2 AMPA receptor-TARP complex elucidated by cryo-EM. Nature 536, 108–111 (2016). 27368053 10.1038/nature18961 PMC4998972

[53] I. G. Denisov, S. G. Sligar, Nanodiscs for the study of membrane proteins. Curr. Opin. Struct. Biol. 87, 102844 (2024). 38795563 10.1016/j.sbi.2024.102844 PMC11283964

[54] S. Q. Zheng, E. Palovcak, J. P. Armache, K. A. Verba, Y. Cheng, D. A. Agard, MotionCor2: Anisotropic correction of beam-induced motion for improved cryo-electron microscopy. Nat. Methods 14, 331–332 (2017). 28250466 10.1038/nmeth.4193 PMC5494038

[55] A. Punjani, J. L. Rubinstein, D. J. Fleet, M. A. Brubaker, cryoSPARC: Algorithms for rapid unsupervised cryo-EM structure determination. Nat. Methods 14, 290–296 (2017). 28165473 10.1038/nmeth.4169

[56] N. R. Voss, C. K. Yoshioka, M. Radermacher, C. S. Potter, B. Carragher, DoG picker and TiltPicker: Software tools to facilitate particle selection in single particle electron microscopy. J. Struct. Biol. 166, 205–213 (2009). 19374019 10.1016/j.jsb.2009.01.004 PMC2768396

[57] J. Zivanov, T. Nakane, B. O. Forsberg, D. Kimanius, W. J. Hagen, E. Lindahl, S. H. Scheres, New tools for automated high-resolution cryo-EM structure determination in RELION-3. eLife 7, e42166 (2018). 30412051 10.7554/eLife.42166 PMC6250425

[58] E. F. Pettersen, T. D. Goddard, C. C. Huang, G. S. Couch, D. M. Greenblatt, E. C. Meng, T. E. Ferrin, UCSF Chimera—A visualization system for exploratory research and analysis. J. Comput. Chem. 25, 1605–1612 (2004). 15264254 10.1002/jcc.20084

[59] P. Emsley, K. Cowtan, Coot: Model-building tools for molecular graphics. Acta Crystallogr. D Biol. Crystallogr. 60, 2126–2132 (2004). 15572765 10.1107/S0907444904019158

[60] P. D. Adams, R. W. Grosse-Kunstleve, L. W. Hung, T. R. Loerger, A. J. McCoy, N. W. Moriarty, R. J. Read, J. C. Sacchettini, N. K. Sauter, T. C. Terwilliger, PHENIX: Building new software for automated crystallographic structure determination. Acta Crystallogr. D Biol. Crystallogr. 58, 1948–1954 (2002). 12393927 10.1107/s0907444902016657

[61] W. F. Borschel, K. A. Cummings, L. K. Tindell, G. K. Popescu, Kinetic contributions to gating by interactions unique to N-methyl-D-aspartate (NMDA) receptors. J. Biol. Chem. 290, 26846–26855 (2015). 26370091 10.1074/jbc.M115.678656 PMC4646337

[62] B. A. Maki, K. A. Cummings, M. A. Paganelli, S. E. Murthy, G. K. Popescu, One-channel cell-attached patch-clamp recording. J. Vis. Exp. , 51629 (2014). 24961614 10.3791/51629 PMC4188217

