# Supplementary material for: Cryo-EM snapshots of NMDA receptor activation illuminate sequential rearrangements
Source: Sci Adv. 2025 Sep 24;11(39):eadx4647. doi: 10.1126/sciadv.adx4647 (PMC12459464; doi:10.1126/sciadv.adx4647)
Supplement: Supplementary file 1 — Figs. S1 to S12 Tables S1 to S7 [file sciadv.adx4647_sm.pdf]

Science Advances
AAAS

Supplementary Materials for

Cryo-EM snapshots of NMDA receptor activation illuminate sequential rearrangements

Jamie A. Abbott *et al.*

Corresponding author: Farzad Jalali-Yazdi, thyzad@gmail.com; Gabriela K. Popescu, popescu@buffalo.edu; Eric Gouaux, gouauxe@ohsu.edu

Sci. Adv. **11**, eadx4647 (2025)
DOI: 10.1126/sciadv.adx4647

This PDF file includes:

Figs. S1 to S12
Tables S1 to S7

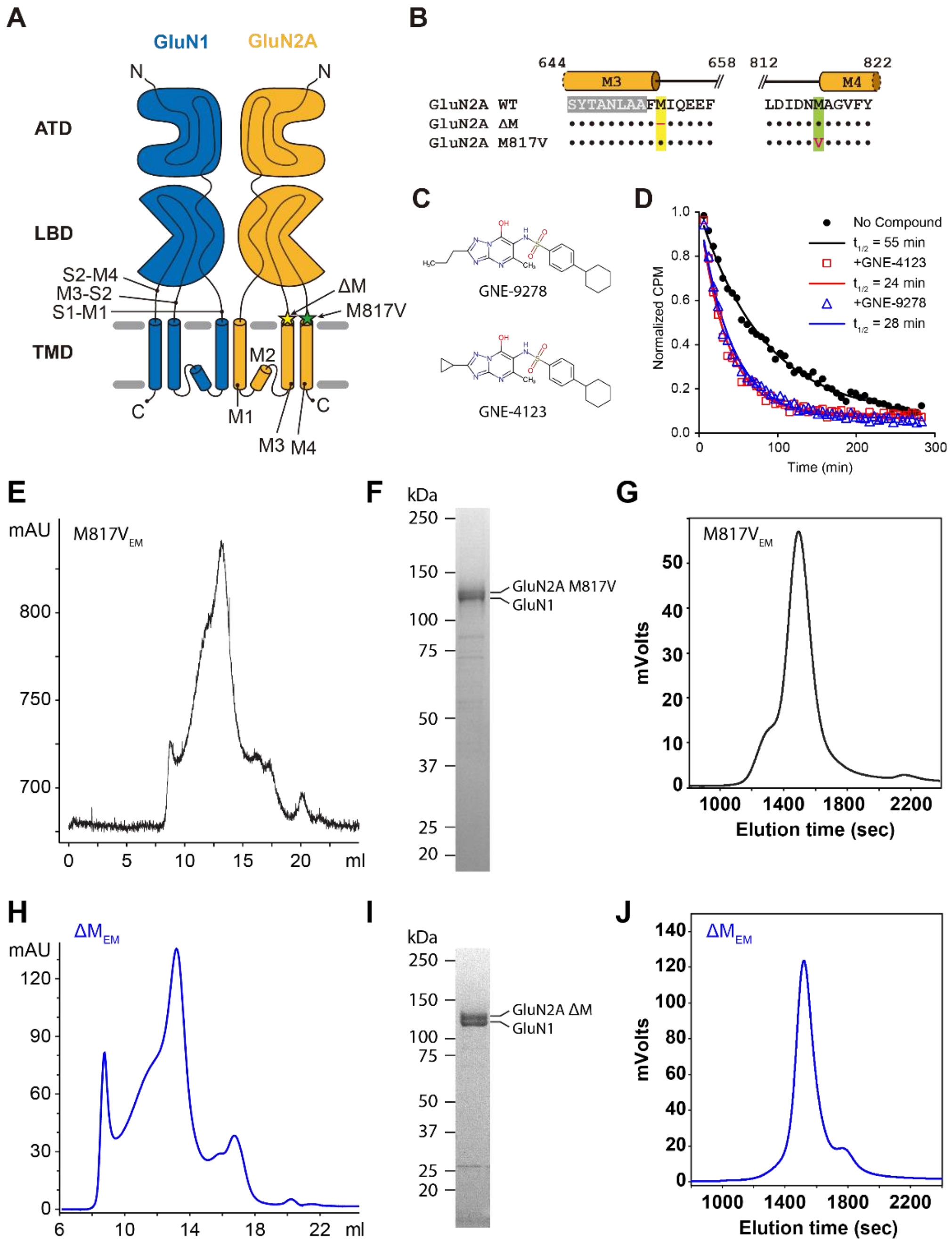

Fig. S1. Biochemical and functional characterization of M817V$_{EM}$ and ΔM$_{EM}$ receptors. (**A**) Cartoon representing GluN1 (blue) and GluN2A (brown) subunits indicates the position of the deleted methionine residue (ΔM) and of the M817V substitution. (**B**) Two segments of the GluN1 sequence indicates the position of the deleted methionine residue (ΔM) and of the M817V substitution. (**C**) Chemical structures of the PAMs used in this study. (**D**) Time-dependent dissociation of [3H]MK-801 (counts per minute) from WT$_{EM}$ proteins in the presence of agonists (Gly/Glu), and the absence or presence of the indicated PAM (symbols) overlayed with single exponential decay functions (lines) fitted to the data. (**E**-**J**) Size-exclusion chromatography (**E** and **H**), SDS-PAGE (**F** and **I**), and fluorescence-detection size-exclusion chromatography (**G** and **J**) results illustrate elution behavior of the M817V$_{EM}$ and ΔM$_{EM}$ proteins.

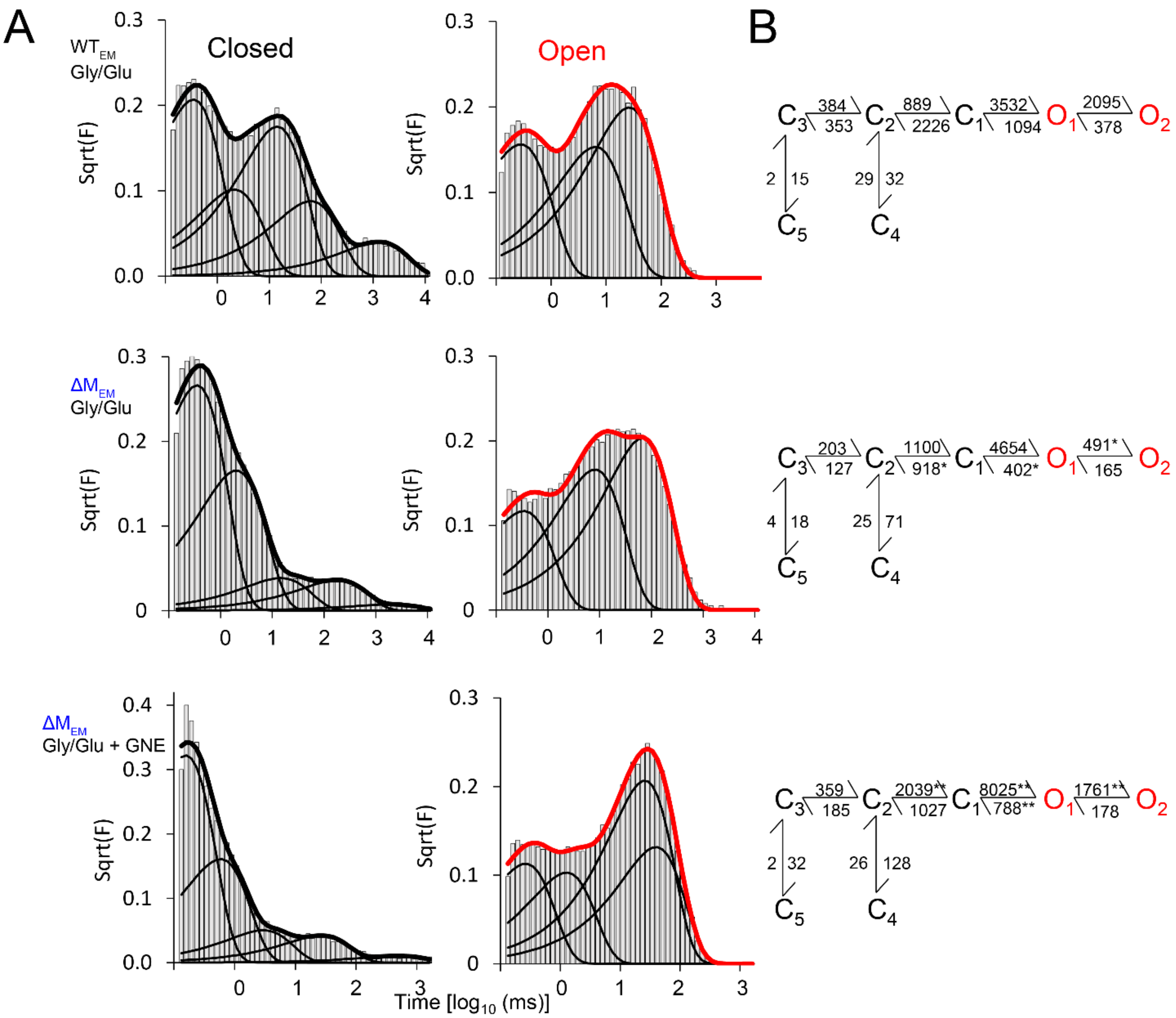

Fig. S2. Kinetic models and dwell time histograms. (A) Exemplary dwell-time histograms of closed (left) and open (right) interval durations detected in one recording with superimposed exponential functions (lines) calculated from a best-fitting model. All files required 5 exponentials for best fit for closed, and at least 2 and up to 4 for open intervals (see Table S3, S4). **(B)** Reaction mechanism derived by fitting files in each data set with the indicated model (5C 2O). Rates are (s^{-1}) represent average values for each set. Significance was determined using t-test Student's unpaired t-test. *,** represent $p < 0.05$ (Student's test) relative to the corresponding rate in WT_{EM} or ΔM_{EM} without GNE models, respectively. The averaged rate constants were used to calculate free energy of transition between states (see Fig. 1D).

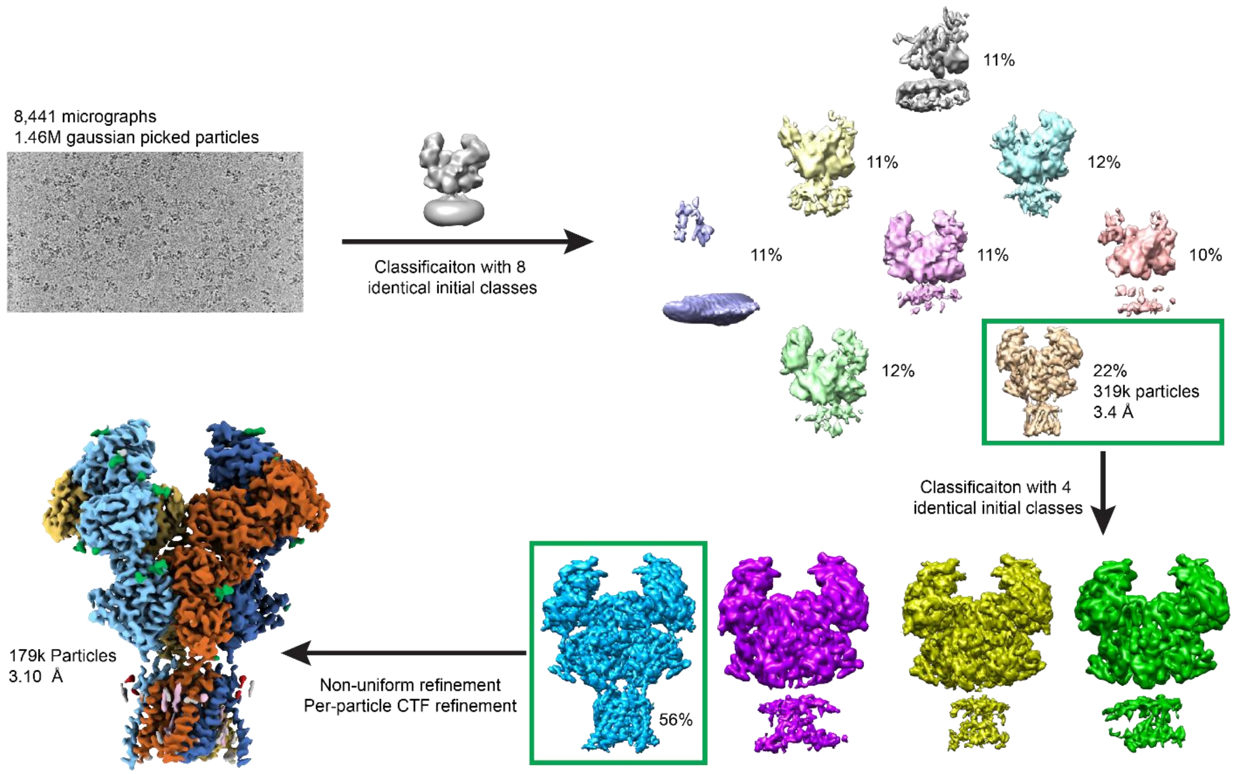

Fig. S3. Single-particle cryo-EM data processing workflow for the glycine/glutamate/GNE-4123-bound open ΔM$_{EM}$ receptors in lipid nanodiscs. A representative micrograph, 3D classification, and reconstruction results are shown. See Methods for the details.

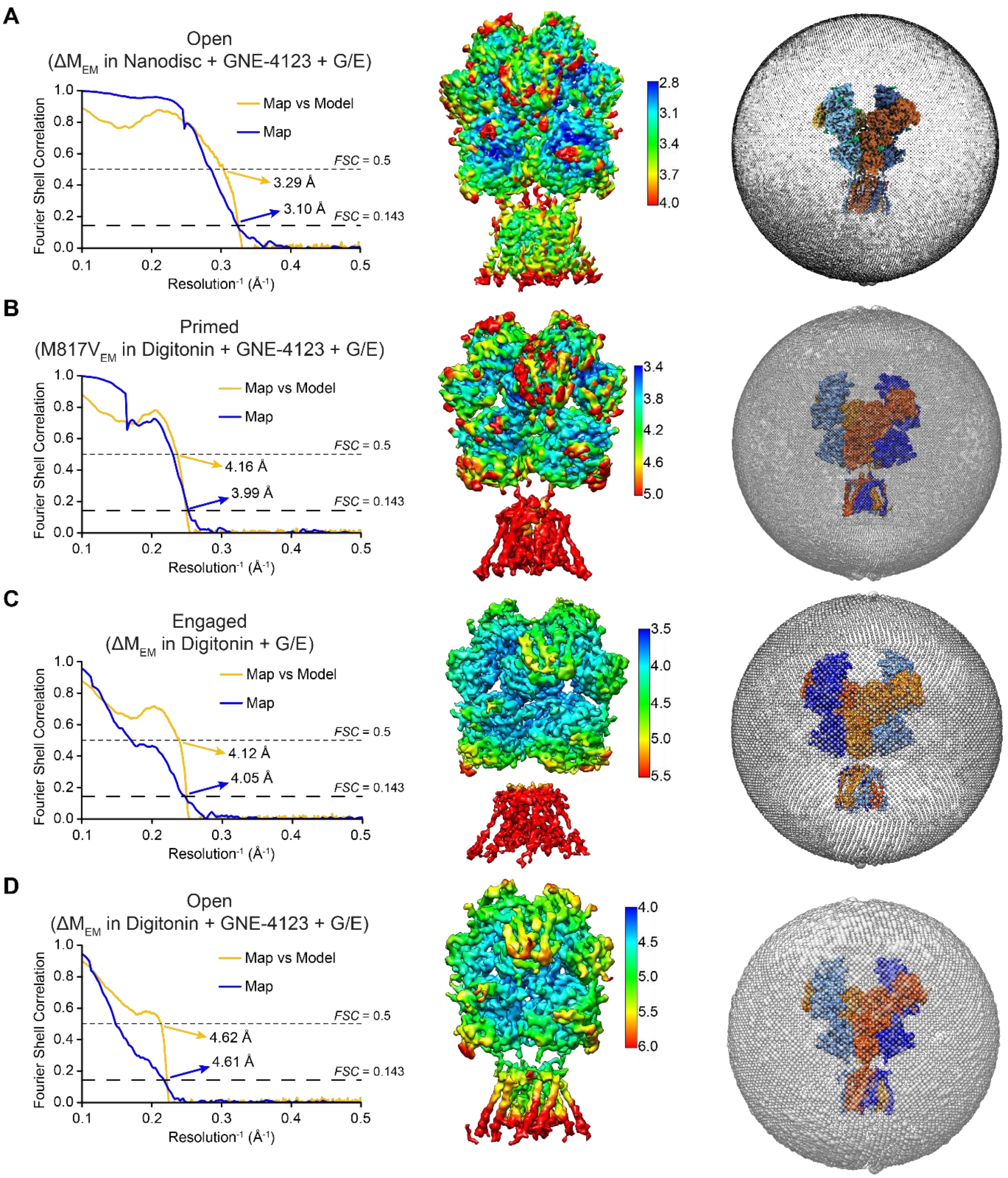

Fig. S4. Single-particle cryo-EM maps and evaluation. (**A-D**) Furrier shell correlation (FSC) curves (left) for the reconstructed maps (blue) and map-to-models (yellow), local resolution estimation (center, units of Å), and orientation distribution (right) of the open ΔM_{EM} receptors in nanodiscs (**A**), the primed $M817V_{EM}$ receptors in digitonin (**B**), the engaged ΔM_{EM} receptors in digitonin (**C**), and the open ΔM_{EM} receptors in digitonin (**D**). All the cryo-EM conditions included the agonists, glycine and glutamate. See Methods for the details.

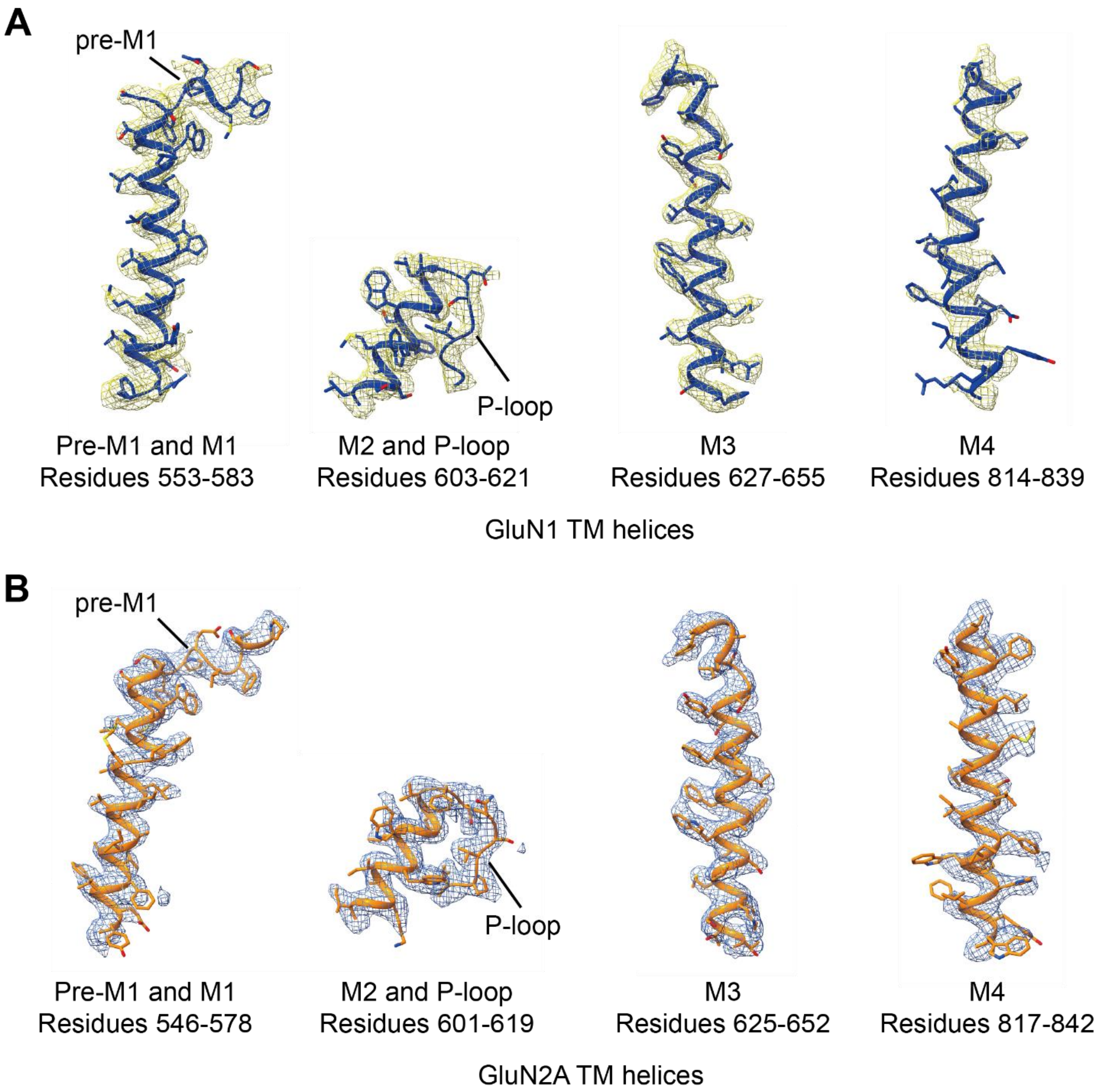

Fig. S5. Map quality of the transmembrane domains. (**A** and **B**) Representative views of the cryo-EM densities for transmembrane helices in GluN1 (**A**) and GluN2A (**B**) subunits from the open ΔM_{EM} receptors in nanodisc, fitted with the molecular models.

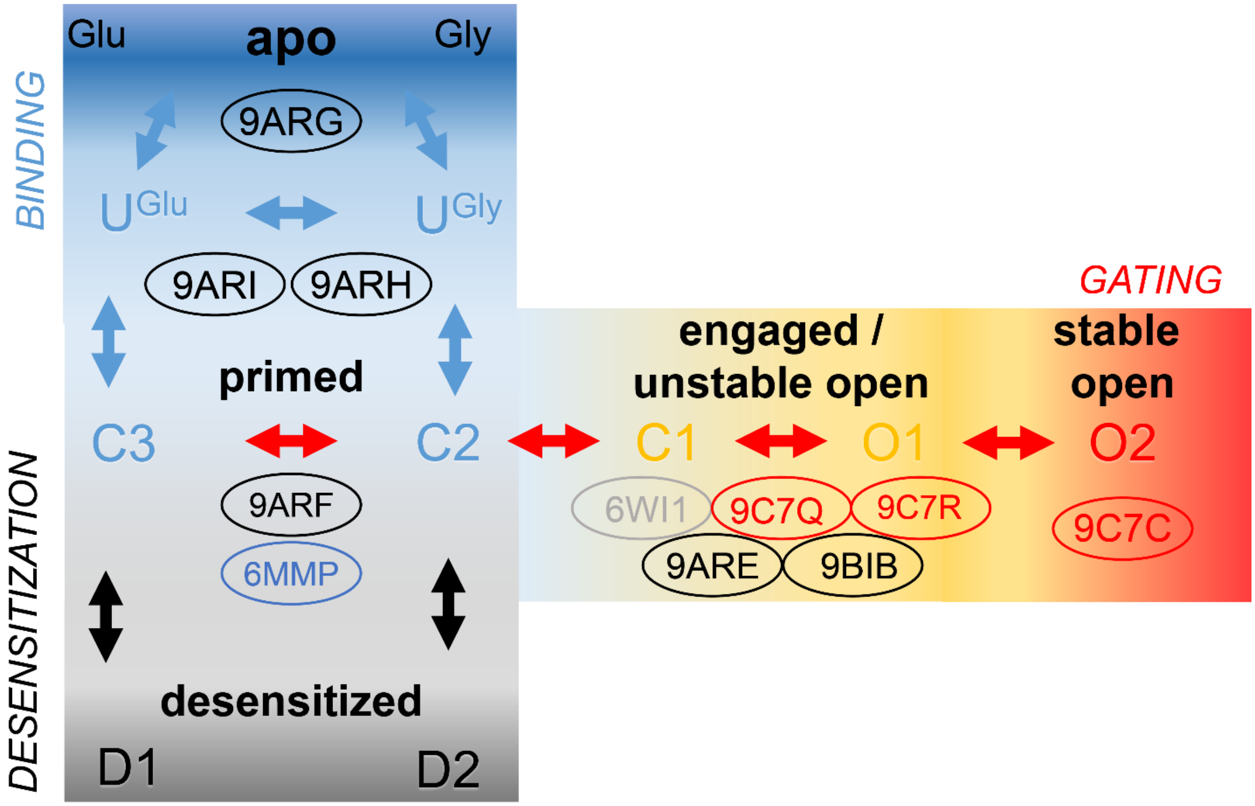

Fig. S6. Reaction mechanism of NMDA receptors and hypothetical correspondence between kinetic states and conformers with known structures. The *activation pathway* consists of sequential *binding* and *gating reactions*. The binding pathway consists of sequential agonist association steps that start with unliganded receptors (apo) and culminate with fully-liganded receptors (primed). Primed receptors can continue along the *activation pathway* with *gating reactions*, which produce short-lived conformations with bent M3 helices (engaged/unstable open) and culminate with stable states that have all four helices bent (stable open). Alternatively, primed states can continue along the *desensitization pathway* into stable closed states (desensitized). Red PDBs are from this paper; black PDBs are from Chou et al., 2024 [22]; blue PDBs are from Jalali-Yazdi et al., 2018 [25]; gray PDBs are from Chou et al., 2020 [41].

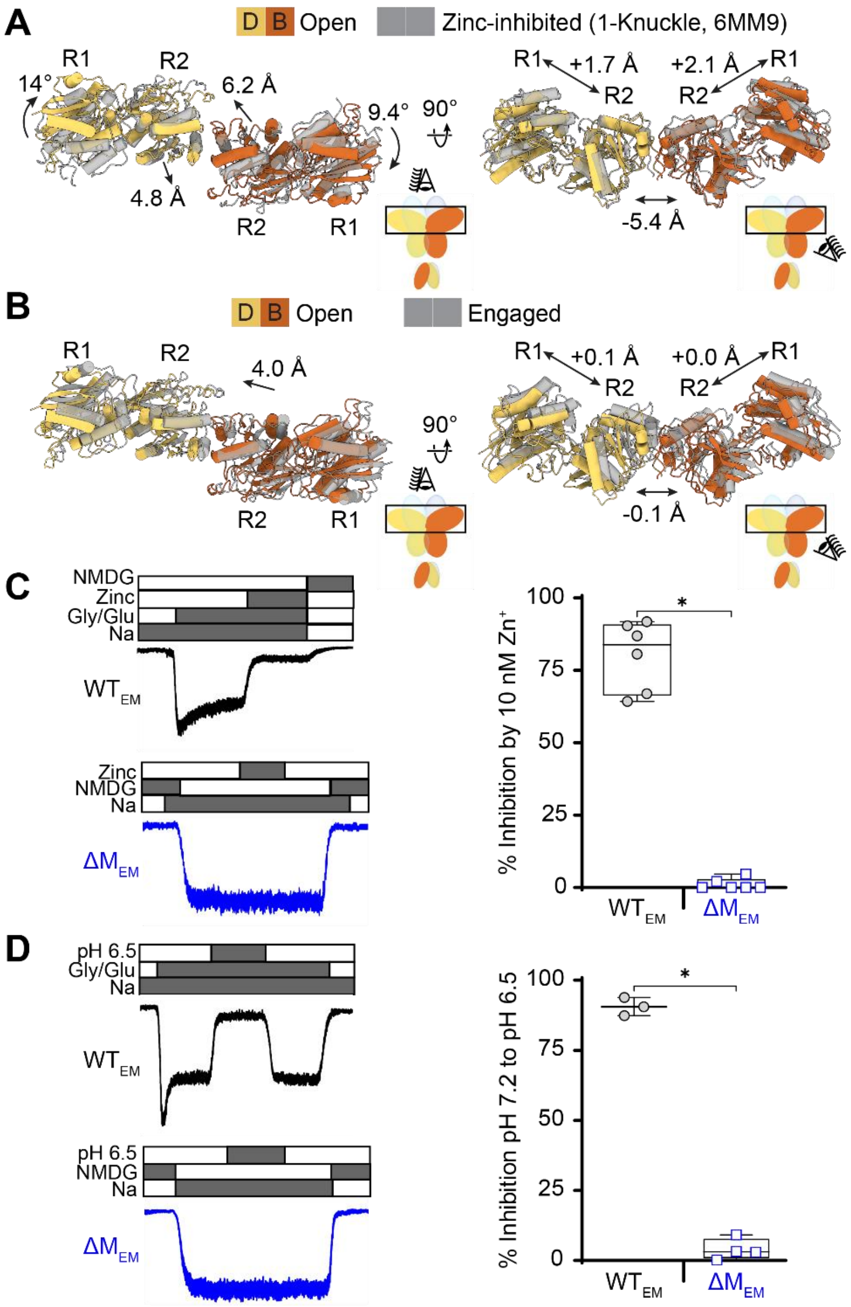

Fig. S7. Conformational changes in ATD layers in different receptor states. (**A**) Structural comparison of the ATD layer in GluN2A subunits from the open (colored) and agonist-bound, zinc-inhibited (PDB code 6MM9, gray) states. (**B**) Structural comparison of the ATD layer in GluN2A subunits from the open (colored) and engaged (gray) states. (**C**) Whole-cell current traces recorded from HEK293 cells expressing WT_{EM} (top, black) and ΔM_{EM} (bottom, black) constructs in the presence of agonists (Gly, Glu), zinc (10 nM), and permeant (Na^{+}) or impermeant ($NMDG^{+}$) ions, as indicated. Bar graph (right) summarizes measured reduction in current relative to basal level (%). (**D**) Whole-cell current illustrates effect of dropping the pH from 7.2 to 6.8 for WT_{EM} (top, black) and ΔM_{EM} (bottom, blue). Bar graph (right) summarizes measured reduction in current at pH 6.8 relative to 7.2 (biological replicates). *, $p<0.0001$ (Student's test).

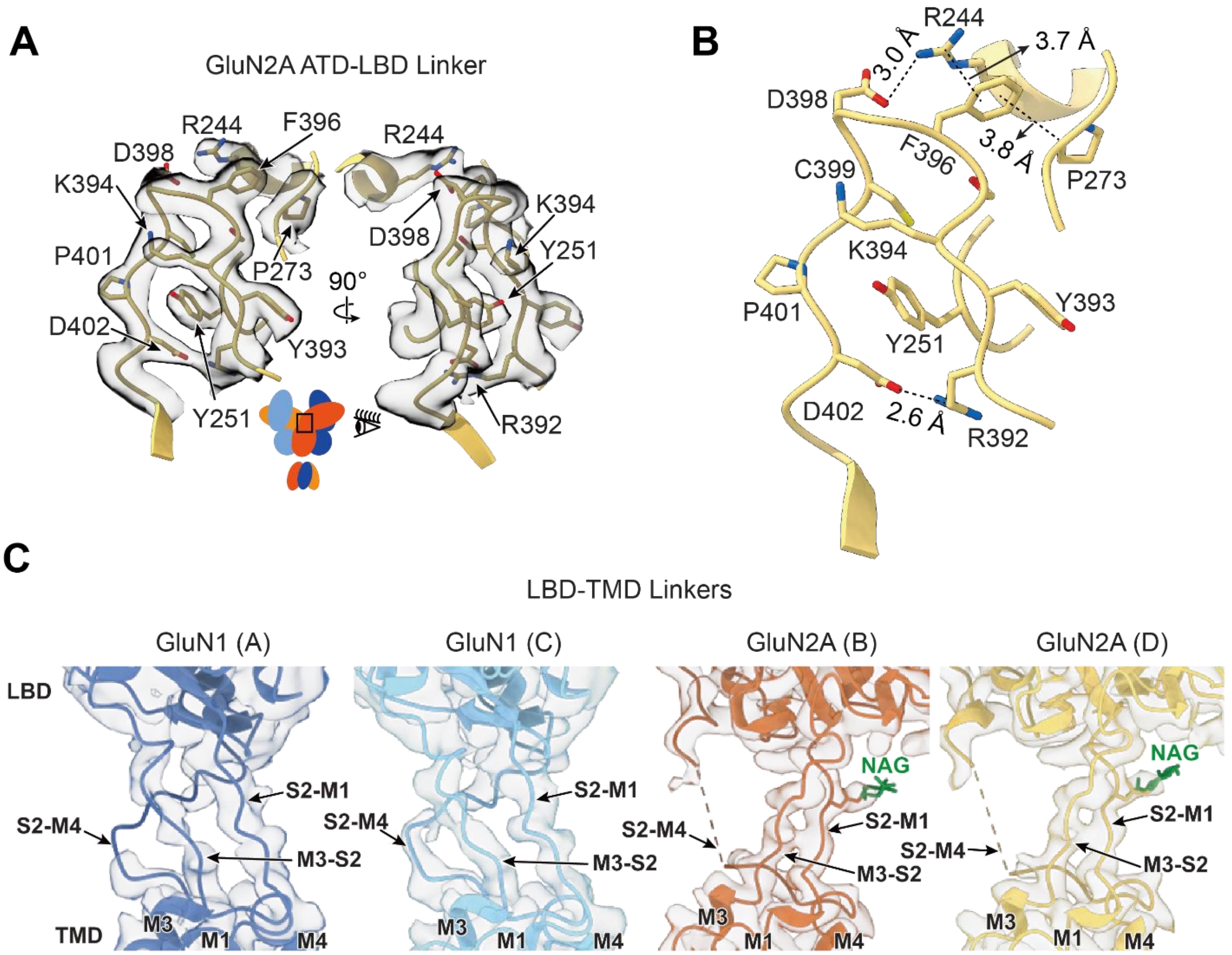

Fig. S8. ATD-LBD and LBD-TMD linkers. (**A**) Cryo-EM density (transparent) and the molecular model of the GluN2A ATD-LBD linker (yellow) from the open ΔM_{EM} receptors in lipid nanodisc. (**B**) The molecular model of the GluN2A ATD-LBD linker with some of the interactions highlighted. (**C**) Cryo-EM densities (transparent) and the molecular models (colored) of the LBD-TMD linkers of the open-pore conformation PDB ID: 9C7C.

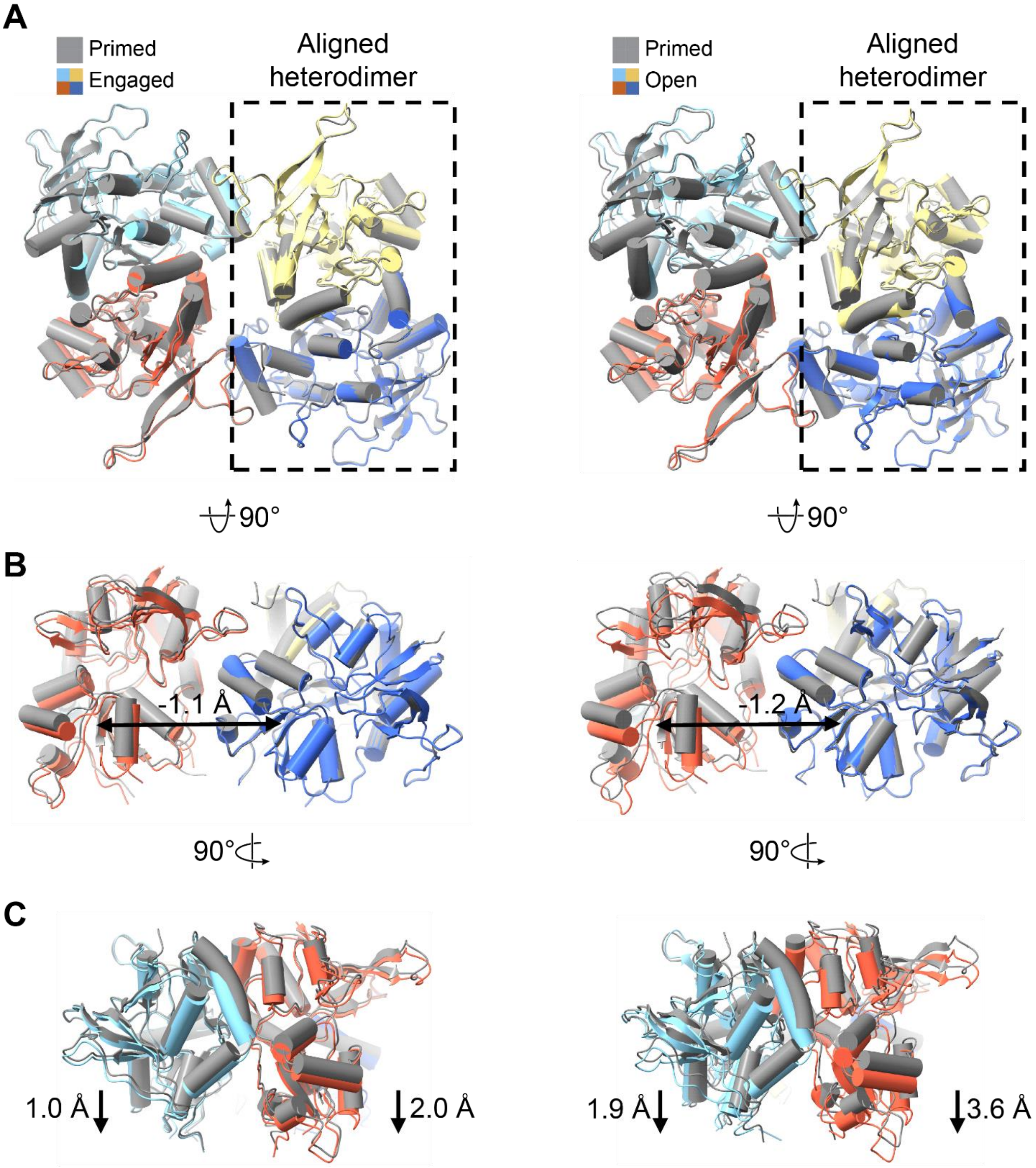

Fig. S9. Structural comparisons of the LBD layers. (**A**) Superposition of the LBD structures of the primed and engaged states (left) and the primed and open states (right), aligned at the GluN1-GluN2A LBD heterodimer (dashed lines) and shown in top view. (**B**) Structural comparisons of the GluN1 and GluN2A LBD dimers reveal decreased distances between the centers-of-mass (COMs) of the D2 lobes in GluN1 and GluN2A during activation. (**C**) The LBD heterodimer on the opposite side moves closer to the transmembrane domain (TMD) upon activation, as shown by the measured changes in COM positions within the D2 lobe of each GluN1 and GluN2A.

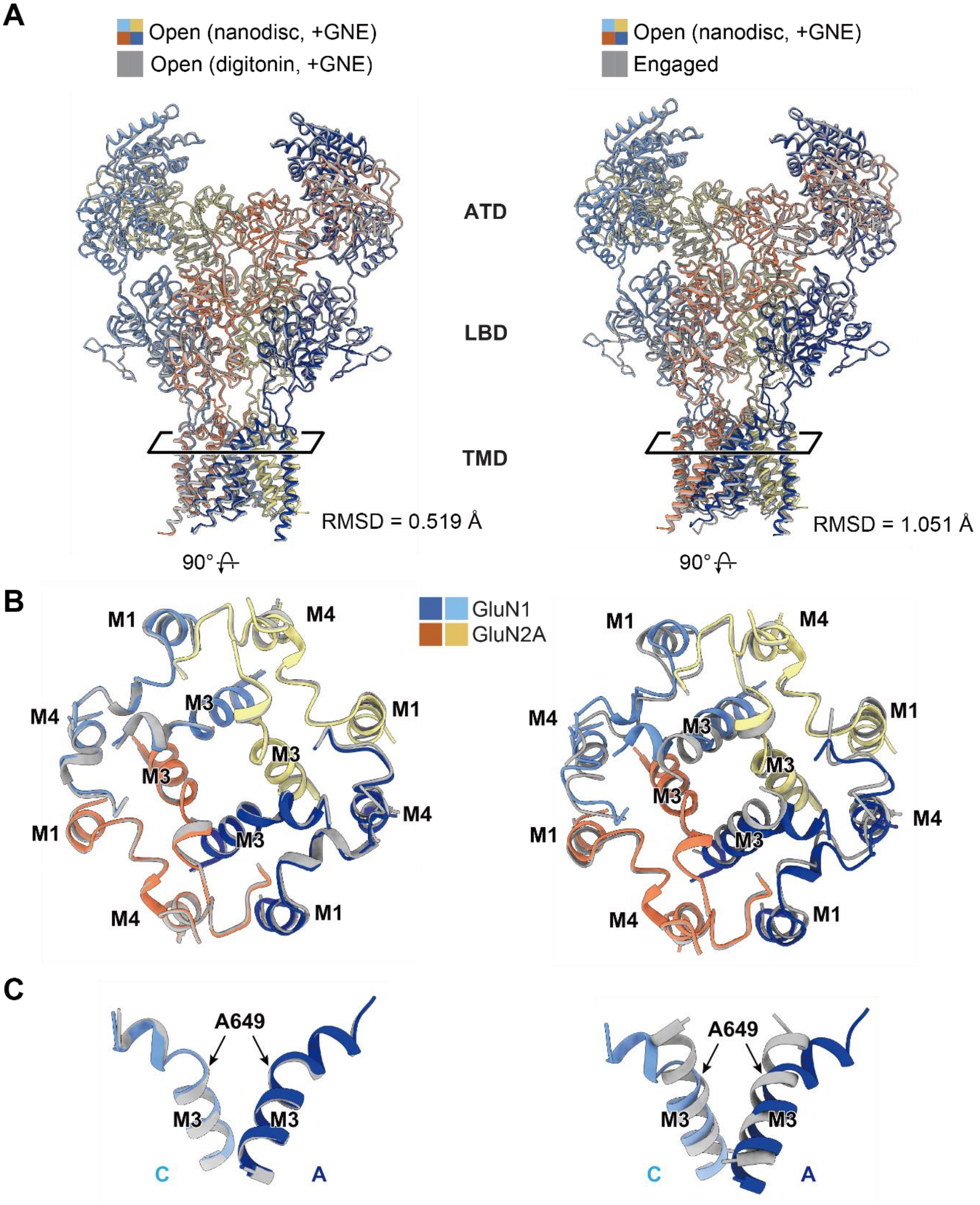

Fig. S10. Structural comparisons for M3-bending in various open-state conformations. (**A**) Structural comparisons of the open-state ΔM_{EM} in lipid nanodisc (colored) with the open-state ΔM_{EM} in digitonin (gray, left) and with the engaged state in digitonin (gray, right). Root-mean-square deviations (RMSD) between the structures are indicated. (**B**) Top views of the channel pores looked at the gate planes in the panel A (squares), demonstrating the conformational differences in M3 segments. (**C**) Side views of the M3 segments of the GluN1 subunits, highlighting the bending of the gating helices is exclusively observed in GNE-4123-bound state.

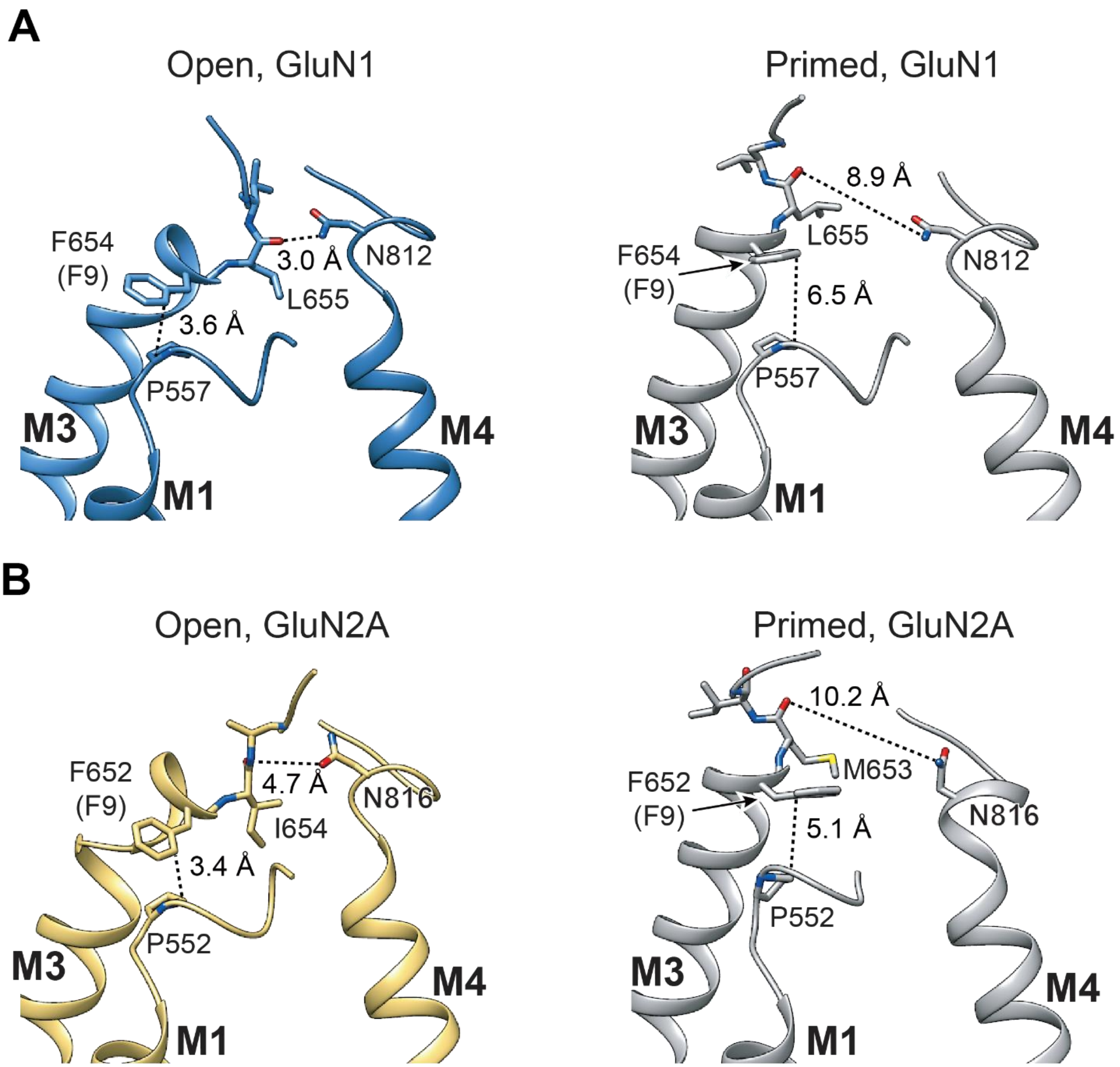

Fig. S11. Additional interactions with the outer gating ring stabilize the bent M3-helix conformation. (**A**) Polarized CH-Pi bonds and H-bonds stabilize the bend M3 helix conformation in GluN1. (**B**) Similar polarized CH-Pi bonds and H-bonds stabilize the bend M3 helix conformation in GluN2A.

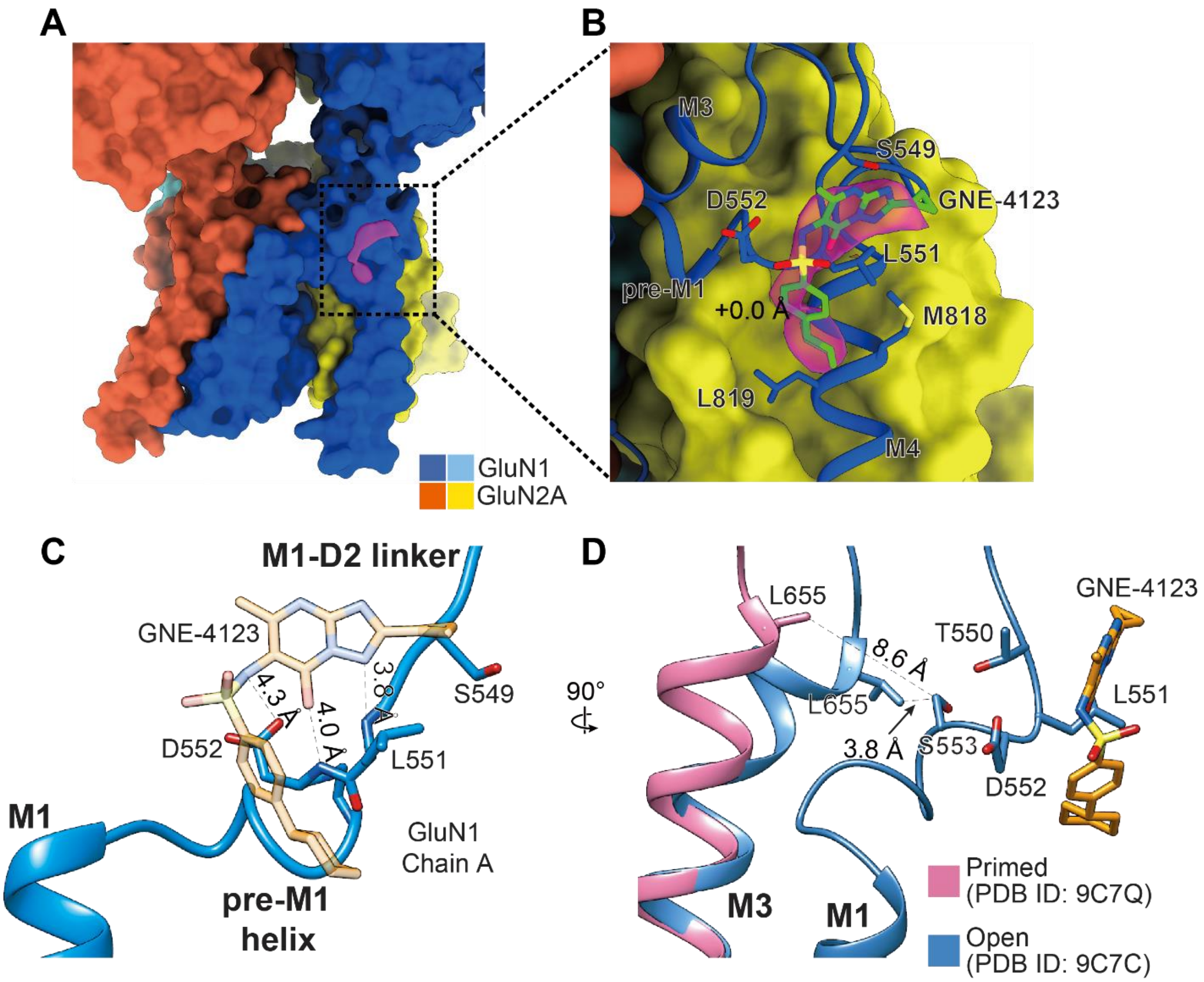

Fig. S12. GNE-4123 binding site on TMD. (**A**) Surface representation of the GNE-4123-bound open-state ΔM_{EM} structure along with the cryo-EM density of GNE-4123 (purple, transparent). (**B**) Zoom-in view showing the GNE-4123 binding pocket with the molecular model and surrounding residues. (**C**) Zoom-in view of the GNE-4123 binding site model. GNE-4123 wraps around L551, with two H-bond interactions forming between the small molecule and amide nitrogens of D522 and L551. (**D**) Comparing the primed and open conformations of the NMDA receptor, both obtained in the presence of GNE-4123. In the open conformation, the kinking of the M3 helix brings L655 closer to the pre-M1 region (stabilized by GNE-4123) by ~5 Å.

Table S1. Kinetics of MK-801 dissociation.

Protein	Micelle	Lipid	Compound	MK-801 $t_{1/2}$ (min)	N
WT_{EM}	digitonin	-	-	42 ± 2	18
WT_{EM}	digitonin	CHS	-	14 ± 1	6
WT_{EM}	digitonin	-	GNE-4123	28 ± 2	6
WT_{EM}	digitonin	-	GNE-9278	29 ± 1	6
$M817V_{EM}$	digitonin	-	-	20 ± 1	4
ΔM_{EM}	digitonin	-	-	8.6 ± 0.3	4
ΔM_{EM}	nanodisc	CHS/BTL	-	3.4 ± 0.3	4

Values are means ± standard error of mean. N are from biological replicates.

Table S2. Single-channel kinetic parameters for WT_{EM} and ΔM_{EM} receptors (pH 8.0).

Receptor	Ligands	*i* (pA)	P_o	MOT (msec)	MCT (msec)	N	Events total	Recording (min)
WT_{EM}	Gly/Glu/--	7.4 ± 0.2	0.44 ± 0.15	7.8 ± 4.3	9.4 ± 2.4	6	231,903	61
ΔM_{EM}	--/--/--	7.1 ± 0.6	0.81 ± 0.07	6.6 ± 3.0	1.3 ± 0.2	5	402,397	45
ΔM_{EM}	Gly/Glu/--	7.4 ± 1.0	0.87 ± 0.03	9.4 ± 2.6	1.4 ± 0.5	6	459,178	70
WT_{EM}	Gly/Glu/ GNE	8.3 ± 1.6	0.48 ± 0.36	14 ± 3	48 ± 61	5	185,546	136
ΔM_{EM}	--/--/GNE	7.6 ± 0.3	0.93 ± 0.01	15 ± 2	1.1 ± 0.2	4	262,966	72

Values are means ± standard deviation. See Methods for full definition of functions. N are from biological replicates.

Table S3. Closed components.

	MCT	τ_{E1} *(ms)*	a_{E1} *(%)*	τ_{E2} *(ms)*	a_{E2} *(%)*	τ_{E3} *(ms)*	a_{E3} *(%)*	τ_{E4} *(ms)*	a_{E4} *(%)*	τ_{E5} *(ms)*	a_{E5} *(%)*
WT_{EM}	9.4 ± 2.4	0.2 ± 0.04	41 ± 4	1 ± 1	27 ± 10	6 ± 3	28 ± 10	73 ± 60	13 ± 23	991 ± 990	1 ± 1
ΔM_{EM}/Gly/Glu	1.4 ± 0.5	0.2 ± 0.1	67 ± 9	1 ± 0.3	28 ± 9	4 ± 2	3 ± 1	36 ± 23	2 ± 1	410 ± 332	1 ± 1
ΔM_{EM}/Gly/Glu/GNE	1.4 ± 0.2	0.1 ± 0.004	71 ± 6	0.5 ± 0.04	25 ± 6	3 ± 1	7 ± 10	46 ± 19	1 ± 1	680 ± 292	0.2 ± 0.1

Values are means ± standard deviation

Table S4. Open components.

	N	events	MOT	τ_{E1} *(ms)*	a_{E1} *(%)*	τ_{E2} *(ms)*	a_{E2} *(%)*	τ_{E3} *(ms)*	a_{E3} *(%)*	τ_{E4} *(ms)*	a_{E4} *(%)*
WT_{EM}	6	231903	7.8 ± 4.3	0.2 ± 0.02	15 ± 5	4 ± 1	29 ± 13	16 ± 8	55 ± 15	----	----
ΔM_{EM}/Gly/Glu	6	459178	9.4 ± 2.6	0.2 ± 0.03	12 ± 3	4 ± 1	35 ± 18	31 ± 14	53 ± 21	----	----
ΔM_{EM}/Gly/Glu/GNE	4	262966	15.3 ± 1.7	0.2 ± 0.02	8 ± 3	1 ± 0.3	9 ± 4	25 ± 10	44 ± 25	51 ± 23	38 ± 30

Values are means ± standard deviation. N are from biological replicates.

Table S5. Equilibrium occupancy of kinetic states.

Receptor	C5	C4	C3	C2	C1	O1	O2
	desensitized		primed		Engaged / Unstable Open		Stable open
WT_{EM}	32.5	6.6	4.2	4.3	2.1	6.3	43.9
ΔM_{EM}	3.8	4.6	0.8	1.4	1.9	22.7	64.7
ΔM_{EM}+GNE	3.0	2.1	0.2	0.4	0.8	8.6	84.9

Table S6. Statistics of cryo-EM data collection and model refinement.

Protein	**M817V$_{EM}$**	**ΔM$_{EM}$**	**ΔM$_{EM}$**	**ΔM$_{EM}$**
PDB code	9C7R	9C7Q	9C7P	9C7C
Label	Primed	Engaged	Open	Open
TM stabilization	detergent micelle	detergent micelle	detergent micelle	nanodisc
Gly/Glu/EDTA (mM)	1/1/2	1/1/2	1/1/2	1/1/2
GNE-4123 (mM)	1	0	0.1	0.1
Data Collection/Processing				
Krios Microscope	PNCC	PNCC	NYSBC	PNCC
Sensor	K3	K3	K2	K3
Voltage (kV)	300	300	300	300
Defocus Range (µm)	-0.8 to -2.0	-0.8 to -2.0	-0.8 to -2.0	-0.8 to -2.0
Exposure time (s)	2.2	2	7.9	1.8
Total Dose (e^-/Å^2)	55	50	50	53
Pixel Size (Å)	0.823	0.826	1.046	0.826
Micrographs	18,873	9,262	3,916	8,048
Particles Processed	3.85M	1.67M	820k	1.46M
Final Particles	431k	165k	133k	179k
Resolution (Å)	3.99	4.05	4.61	3.1
Symmetry	C1	C1	C1	C1
Molecular Model				
Model Resolution (Å)	4.2	4.1	4.6	3.3
Model composition				
Non-hydrogen atoms	25,867	25,046	25,132	26,221
Protein residues	3,173	3,166	3,178	3,184
Ligands	55	0	0	60
R.m.s. deviations				
Bond lengths (Å)	0.003	0.003	0.003	0.003
Bond angles (°)	0.606	0.561	0.612	0.629
Validation				
MolProbity score	1.71	1.73	1.83	1.72
Clash score	9.22	9.58	12.73	9.11
Rotamer outliers (%)	0.04	0.00	0.00	0.11
Cβ outliers (%)	0.00	0.00	0.00	0.00
Ramachandran plot				
Favored (%)	96.57	96.50	96.68	96.49
Allowed (%)	3.43	3.50	3.32	3.51
Disallowed (%)	0.00	0.00	0.00	0.00
CC (mask)	0.84	0.84	0.79	0.83

Table S7. Effects of ligands on macroscopic current amplitude.

		WT_{EM}		ΔM_{EM}	
Modulator	Conc. (mM)	% change	n	% change	n
Mg	1.0	-96 ± 2	5	-94 ± 3	7
AP5	0.2	-83 ± 5	6	-1 ± 2*	6
7-CKA	0.1	-97 ± 2	7	-76 ± 8*	5
pH	6.5	-91 ± 3	3	-4 ± 4*	4
Zinc	$1x10^{-5}$	-80 ± 12	6	-1 ± 2*	5
GNE-9278	0.05	+128 ± 49	10	+33 ± 24*	10

*Values are means ± standard deviation. *Relative to WT_{EM} adjusted p-value <0.0001 (Student's test). See Methods for description of statistical analysis. N are from biological replicates.*
